# Recent applications of neural interfaces in musculoskeletal disorders: from rehabilitation to regeneration

**DOI:** 10.3389/fbioe.2026.1964470

**Published:** 2026-10-27

**Authors:** Xudong Zhao, Keyi Chen, Bicheng Wang, Jinquan Ma, Zixi Wang, Chenfei Gao, Tenghui Zhang, Fazhi Zang, Bo Hu, Huajiang Chen

**Affiliations:** Spine Center, Department of Orthopedics, Changzheng Hospital, Naval Medical University, Shanghai, China

**Keywords:** bioelectronic medicine, closed-loop neuromodulation, musculoskeletal disorders (MSDs), neural interfaces, Orthopedics

## Abstract

Musculoskeletal disorders (MSDs) represent a primary cause of global long-term disability, stemming from musculoskeletal tissue damage or impaired neural motor and homeostatic regulation. The musculoskeletal system participates in a bidirectional neuro-musculoskeletal-immune network, explaining why conventional tissue repair and mechanical rehabilitation often fail to fully restore tissue and motor function. Neural interface (NI)-based bioelectronic medicine provides a precise, translational strategy to reconstruct disrupted neural commands, remodel abnormal sensory pathways, and regulate local inflammation and metabolism for MSD recovery. This review comprehensively summarizes recent NI applications in diverse MSDs, including structural injuries and degenerative diseases, as well as functional deficits induced by spinal cord injury, stroke and peripheral nerve damage. We highlight the clinical transition from open-loop stimulation and passive assistive devices to closed-loop adaptive neuromodulation systems for motor control, sensory restoration and pain relief, alongside emerging bioelectronic regenerative strategies. We further discuss advanced NI material and system designs, covering multimodal sensing, low-latency neuromorphic computing and energy-autonomous modules. Key translational barriers and a roadmap for next-generation regenerative MSD therapies are finally outlined.

## Background

Musculoskeletal disorders (MSDs) remain a leading cause of long-term disability and diminished quality of life worldwide (Global and regional, 2023). Broadly, MSDs fall into two categories: the first involves direct damage to musculoskeletal tissues, where inadequate healing compromises the structural support required for movement (Long et al., 2022); the second is characterized by dysfunction in the nervous system controlling the musculoskeletal system such as spinal cord injury (SCI), where the loss of motor signals precludes motor function (Global, 2023).

Despite significant advances in regenerative medicine, tissue engineering, and rehabilitation technologies, substantial clinical needs remain unmet. For instance, even with intensive rehabilitation, most patients with SCI fail to recover independent walking or hand dexterity. Similarly, chronic back pain secondary to intervertebral disc degeneration often responds poorly to medication or surgery, resulting in long-term reliance on analgesics and a deteriorating quality of life (Knezevic et al., 2021). Furthermore, bone and cartilage repair is frequently hindered by inflammation, inadequate vascularization, and mechanical instability (Li et al., 2026). Even after anatomical repair of muscles or ligaments, they often exhibit persistent functional deficits and a high risk of re-injury (Kotian et al., 2026). Collectively, these challenges highlight a critical reality: isolated tissue repair or mechanical rehabilitation alone is insufficient to fully restore functional integrity of the neuromusculoskeletal system.

Traditionally, the musculoskeletal system is viewed as a passive effector, operating under the unidirectional command of motor neurons. However, this paradigm is increasingly being challenged by recent advances in systematic biology, single-cell sequencing and optogenetics over the past decade. Emerging evidence, particularly from the perspective of interoception, reveals the musculoskeletal system as a highly dynamic endocrine and immunoregulatory organ, which is critically engaged in intricate and bidirectional crosstalk with the nervous system (Severinsen and Pedersen, 2020; Xu et al., 2025a; Lv et al., 2022; Yang et al., 2024; Xiao et al., 2023). Disruption of homeostasis within this “neuro-musculoskeletal-immune” network has been increasingly recognized as a central driver in the pathogenesis of MSDs. Consequently, therapies targeting bone or muscle tissues alone may yield limited efficacy. Instead, re-establishing normal communication between the nervous system and these target organs represents a pivotal frontier for next-generation interventions.

The emergence of bioelectronic medicine, represented by neural interface technology, provides new tools to intervene in this complex network (Rana et al., 2025; Hettick et al., 2025). Unlike traditional pharmacotherapies, NIs can modulate specific neural circuits with millisecond temporal resolution and micrometer-scale spatial precision (Sahasrabudhe et al., 2025; Ibáñez et al., 2025; Farina et al., 2023; Won et al., 2020). Beyond merely substituting for lost motor commands, these interfaces can regulate local metabolic microenvironments and immune responses by simulating endogenous bioelectric signals, thereby facilitating tissue homeostasis and regeneration.

Accordingly, this review aims to systematically examine the recent advances and transformative potential of neural interface technologies in the management of MSDs by first elucidating the physiological interactions within the neuro-musculoskeletal system, then outlining how the nervous system maintains homeostasis and motor function, and finally discuss specific application strategies for NIs across a spectrum of conditions ranging from structural pathologies such as spinal, articular, soft tissue and metabolic bone disorders to functional deficits like SCI. In addition, we specifically address innovations in next-generation hardware architectures by focusing on how integration of neuromorphic computing enables edge processing and self-decision making regarding pathological signals for the sake of establishing an integrated “sensing-computing-modulation” closed-loop system. Finally, we provide a summary of current technical challenges and propose a perspective on future directions in this field.

This narrative review was developed through searches of PubMed, Web of Science, and Google Scholar, with a primary focus on literature published between 2020 and 2026, while earlier seminal studies were included where relevant. Search strings combined terms for neural interfaces and bioelectronic medicine with terms for musculoskeletal disorders, neuromodulation, regeneration, closed-loop control, sensing, materials, and rehabilitation. We included peer-reviewed original research articles, clinical studies, and relevant review articles published in English. Publications were excluded if they were not directly relevant to the scope of neural interfaces and musculoskeletal disorders, lacked sufficient methodological or mechanistic detail, or represented non-peer-reviewed or preliminary reports without adequate supporting evidence. Representative studies were prioritized according to clinical relevance, mechanistic contribution, technical novelty, recency, and level of evidence, with particular emphasis on studies that illustrated distinct therapeutic targets, interface strategies, or translational challenges.

## Mapping the integrated crosstalk of the neuro-musculoskeletal system

As outlined in the background, bidirectional crosstalk within the neuro-musculoskeletal system is fundamental to maintaining tissue homeostasis and motor function. To develop effective NIs, it is essential to first decode this complex biological communication network. Modern research in systematic biology has now provided a detailed mapping of this network, revealing that the musculoskeletal system transcends its traditional role as a mere mechanical effector; rather, it functions as an integrated complex that coordinates mechanotransduction, endocrine regulation, and immune surveillance.

### Structural crosstalk of the neuro-immune-metabolic nexus

#### The central nervous system (CNS) and musculoskeletal homeostasis

Bone metabolic homeostasis is not an isolated, local phenomenon but a systemic process strictly orchestrated by the hypothalamus–sympathetic/parasympathetic–bone axis (the “brain–bone axis”). Recent research has delineated the hierarchical architecture of this axis with unprecedented precision, tracing pathways from central command encoding to peripheral execution. Through this intricate regulation, the nervous system maintains bone mass via a delicate equilibrium; conversely, disruption of this balance constitutes a fundamental pathological basis for osteoporosis and structural bone degeneration.

As the “central commander” of bone metabolism, the hypothalamus exhibits remarkable nuclear specificity and functional complexity (Shi and Chen, 2024). Specifically, the ventromedial hypothalamic nucleus (VMH) and the arcuate nucleus (ARC) constitute two distinct, counterbalancing nodes within this regulatory network. Research has identified SF1-positive neurons in the VMH as a critical negative regulator. While the activation of these neurons significantly boosts systemic energy expenditure, it paradoxically results in a reduction of bone mineral density. This phenomenon unveils a distinct central mechanism of “energy–bone uncoupling”, suggesting that under certain physiological conditions, the organism may sacrifice bone mass to prioritize elevated metabolic rates (Idelevich et al., 2018; Liang et al., 2025). In contrast, AgRP and POMC neurons residing in the ARC exert an osteogenic effect. They stimulate systemic bone formation by releasing the neurotransmitter galanin via the activator protein-1 (AP1) signaling pathway (Zhang et al., 2025a). This underscores that the hypothalamus does not merely issue a monolithic command for “growth” or “resorption”; rather it exerts a precise, and sometimes antagonistic control over the skeleton through the combinatorial activity of distinct nuclei. These central commands are ultimately relayed to the bone microenvironment via the autonomic nervous system, manifesting as an intricate interplay between sympathetic and parasympathetic regulation. The sympathetic nervous system predominantly assumes a “catabolic” role. Norepinephrine (NE) released from sympathetic terminals exerts a dual inhibitory effect. On one hand, it directly arrests osteoblast proliferation via β2-adrenergic receptors (β2-ARs) expressed on these cells, and on the other hand it concurrently stimulates the secretion of RANKL. The latter mechanism potentiates osteoclast activity, thereby accelerating bone resorption (Elefteriou, 2018; Zhong and Xia, 2021).

In stark contrast, the parasympathetic nervous system confers osteoprotection via the “cholinergic anti-inflammatory pathway”. Acetylcholine released upon vagal activation targets the α7 nicotinic acetylcholine receptor (α7nAChR), significantly suppressing the release of pro-inflammatory cytokines and thereby indirectly impeding inflammation-induced bone resorption (Xia et al., 2025a). Moreover, central IL-1 signaling has been shown to obligatorily rely on parasympathetic relay to the skeleton to maintain normal bone mass (Bajayo et al., 2012). This dynamic equilibrium between sympathetic and parasympathetic drives serves as the cornerstone of bone homeostasis; once this balance is tipped, for instance by sympathetic overactivity, the skeletal system rapidly shifts towards a state of negative remodeling balance (Brazill et al., 2019).

Aberrant remodeling of the central neural circuits under pathological conditions often acts as the initiating driver of bone loss, a phenomenon particularly evident in stress-induced osteoporosis. Zhao and colleagues meticulously delineated the neuroanatomical basis underlying this process, revealing that disruption of the equilibrium between the central amygdala (CeA) and the locus coeruleus (LC) precipitates the hyperactivation of LC noradrenergic neurons. This stress signal is propagated to the paraventricular nucleus of the hypothalamus (PVH), ultimately driving sustained peripheral sympathetic tone that directly fuels bone loss (Zhao et al., 2025). Furthermore, Huo et al. demonstrated that CRH neurons in the PVH project polysynaptically to the articular synovium, where they release NE to act on PRG4+ progenitors. This pathway actively suppresses cartilage regeneration while promoting fibrosis, suggesting that under specific stress conditions, the CNS engages a “regenerative brake” mechanism to limit tissue repair (Huo et al., 2026).

Beyond direct electrochemical signaling, a groundbreaking study by Chen et al. identified extracellular vesicles (EVs) as a novel material vector for “brain-bone” communication (Chen et al., 2026a). They discovered that senescent neurons, particularly in the hippocampus and cortex, secrete WDFY1-loaded vesicles. These EVs traverse the blood–brain barrier (BBB) to specifically target bone marrow stromal cells (BMSCs), whereby they disrupt Retromer complex function, thus inhibiting osteogenesis and favoring adipogenesis. This finding extends the current framework in that it provides the first physical evidence that brain ageing can be materially “transmitted” to the skeleton, driving osteoporosis and marrow adiposity. This not only broadens our understanding of structural bone pathologies but also highlights the blockade of specific neurogenic EVs as a potential therapeutic strategy for age-related osteoporosis. Completing this bidirectional loop, peripheral signals also modulate central function: skeletal muscle-derived irisin and cathepsin B can cross the BBB to remodel cognition, while bone-derived osteocalcin modulates memory via the GPR158 receptor (Figure 1a) (Kostka et al., 2024; Li J. et al., 2025).

**FIGURE 1:**
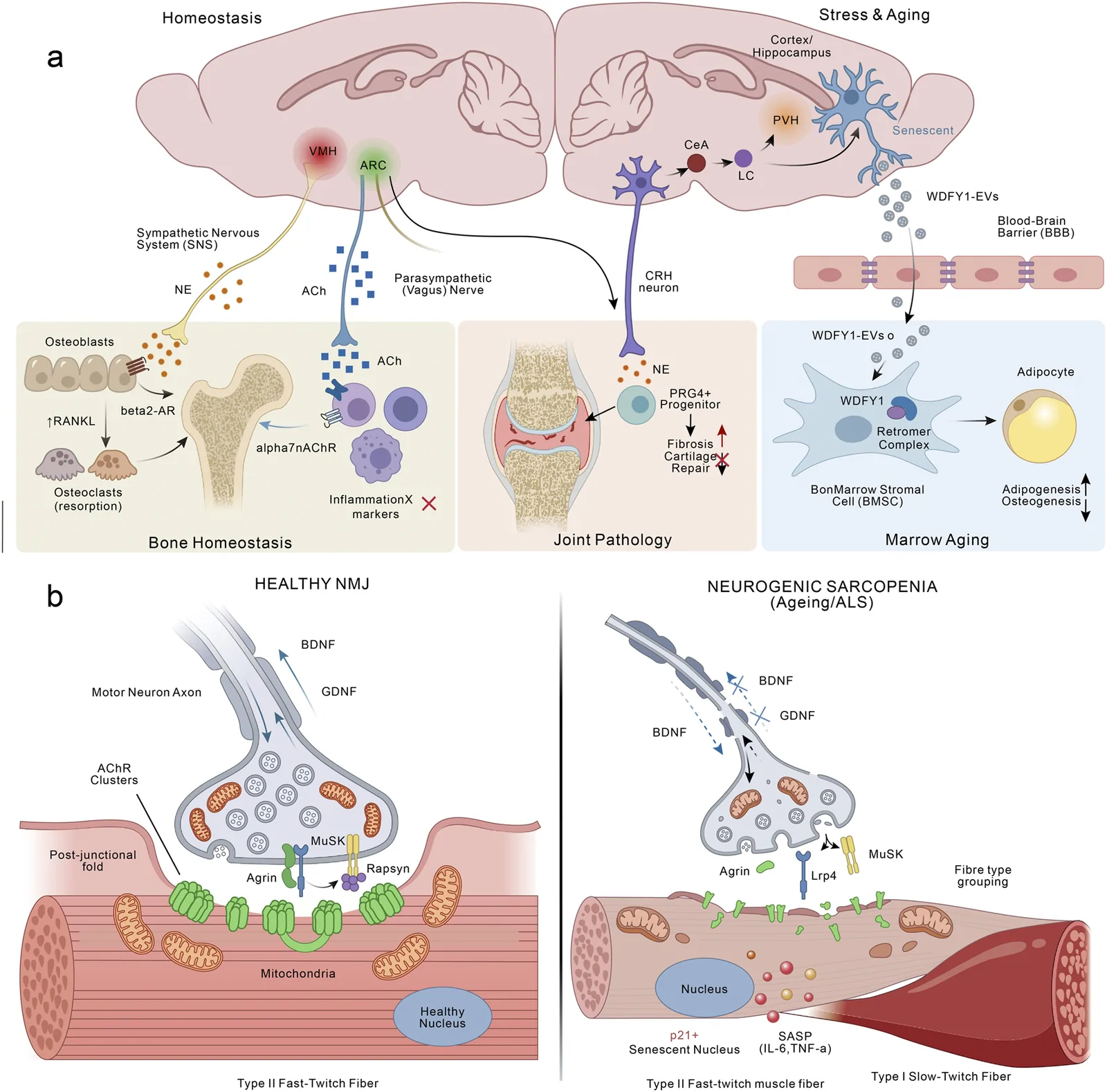
Neural control of musculoskeletal homeostasis and neurogenic sarcopenia. **(a)** VMH/ARC autonomic outputs regulate bone: sympathetic NE acting on osteoblast β2-AR increases RANKL-driven resorption, while vagal ACh suppresses inflammatory cues via α7nAChR. Stress heightens sympathetic tone by remodeling the CeA–LC–PVH circuit; PVH CRH-neuron pathways promote fibrosis and inhibit cartilage repair by acting on PRG4+ progenitors. WDFY1-EVs released by ageing senescent cortex/hippocampus cross the BBB to BMSCs, disrupting the retromer complex, reducing osteogenesis and increasing adipogenesis. **(b)** Healthy NMJ maintains AChR clusters via Agrin–Lrp4–MuSK; ageing/ALS shows denervation, endplate fragmentation, p21+ senescent nuclei with SASP and fibre-type grouping. Abbreviations: VMH ventromedial hypothalamus, ARC arcuate nucleus, PVH paraventricular hypothalamic nucleus, CeA central amygdala, LC locus coeruleus, SNS sympathetic nervous system, NE norepinephrine, ACh acetylcholine, RANKL receptor activator of nuclear factor kappa-B ligand, β2-AR beta-2 adrenergic receptor, α7nAChR alpha7 nicotinic acetylcholine receptor, CRH corticotropin-releasing hormone, PRG4 proteoglycan 4, WDFY1 WD repeat and FYVE domain containing 1, EVs extracellular vesicles, BBB blood–brain barrier, BMSC bone marrow stromal cell, NMJ neuromuscular junction, ALS amyotrophic lateral sclerosis, BDNF brain-derived neurotrophic factor, GDNF glial cell line-derived neurotrophic factor, AChR acetylcholine receptor, MuSK muscle-specific kinase, Lrp4 low-density lipoprotein receptor-related protein 4, SASP senescence-associated secretory phenotype, IL-6 interleukin-6, TNF-α tumor necrosis factor-alpha.

#### Peripheral nervous system and musculoskeletal homeostasis

##### Neuromuscular junction and skeletal muscle degeneration

Against the backdrop of global population ageing, the age-related loss of skeletal muscle mass and function, termed sarcopenia, has emerged as a formidable public health challenge. While conventionally attributed to intrinsic metabolic defects within muscle cells, such as mitochondrial dysfunction or impaired protein synthesis, a series of breakthrough studies in recent years has established the concept of “Neurogenic Sarcopenia”. This paradigm posits that the structural remodeling and functional decline of the neuromuscular junction (NMJ) are not merely consequences of muscle atrophy, but rather the primary upstream mechanisms that initiate and perpetuate this pathological process (Kupriyanova et al., 2025). The NMJ, as a specialized synapse between motor neuron terminals and muscle fiber membranes, exhibits a highly folded topology in healthy adults. This architecture maximizes the synaptic contact area, ensuring high-fidelity acetylcholine (ACh) transmission. However, in models of ageing and neurodegenerative diseases such as ALS, the NMJ underwent profound morphological degeneration, characterized by postsynaptic endplate fragmentation, presynaptic denervation, and acetylcholine receptor (AChR) cluster dispersion within the synaptic cleft (Arnold and Clark, 2023; Fujitani et al., 2025).

Recent investigations in the SOD1G93A rat model of ALS have unveiled a pathological phenomenon termed the “dying-back mechanism” (Eckardt et al., 2025). Through gene silencing techniques, researchers demonstrated that motor neuron degeneration did not commence at the soma in the spinal cord anterior horn but rather at the axon terminals. This implies that the compromise of NMJ integrity is a precursor to, rather than a sequel of, neuronal death. Research at the molecular level has shown that Bet1L, a SNARE protein involved in vesicle transport, plays a critical role in NMJ maintenance, and that its depletion precipitates early synaptic disassembly, which further corroborates the dominance of presynaptic pathology. Furthermore, the Agrin-Lrp4-MuSK-Dok7 axis serves as the canonical pathway for maintaining NMJ structure (Tintignac et al., 2015). Physiologically, Agrin secreted by nerve terminals activates MuSK receptors on the sarcolemma, promoting AChR clustering. In ageing muscle, however, the signaling efficiency of this pathway is markedly compromised. Reduced MuSK autophosphorylation directly triggers the disassembly of the postsynaptic apparatus.

Additionally, the exorbitant energetic demands of the synaptic region render it uniquely susceptible to mitochondrial quality control failures (Kupriyanova et al., 2025). Emerging evidence indicates that the local accumulation of reactive oxygen species (ROS) destabilizes cytoskeletal proteins. Concurrently, impaired mitochondrial biogenesis and defective mitophagy in both pre- and postsynaptic compartments act as key drivers of NMJ mechanical fragility. The degradation of synaptic matrix proteins, such as laminin and collagen Q—which are indispensable for anchoring acetylcholinesterase (AChE) and maintaining synaptic alignment—further exacerbates this instability with age (Moss et al., 2025).

The neurogenic injury resulting from these NMJ anomalies exhibits remarkable specificity of preferential denervation of fast-twitch (Type II) fibers. To compensate for this loss of innervation, adjacent slow-twitch (Type I) motor neurons execute compensatory collateral sprouting to re-innervate the orphaned fibers. This remodeling results in a characteristic histological phenomenon known as “fiber type grouping”, which drives a global phenotypic shift towards a slow-oxidative profile. This micro-level mechanism provides an elegant explanation for a distinct clinical feature of the elderly: the decline in explosive power (governed by Type II fibers), which typically precedes the loss of endurance (governed by Type I fibers).

High-throughput sequencing technologies, specifically single-nucleus RNA sequencing (snRNA-seq), have further refined our resolution of this process (Kupriyanova et al., 2025). These studies have unveiled significant “nuclear heterogeneity” within single muscle fibers. In ageing muscle, nuclei within Type II fibers exhibit a robust proteolytic signature, which may explain their propensity for atrophy. Conversely, nuclei in Type I fibers display a transcriptional reprogramming towards glycolytic metabolism. Critically, researchers have identified a distinct population of senescent nuclei expressing high levels of *CDKN1A* (p21). These nuclei not only suffer functional decline but also secrete Senescence-Associated Secretory Phenotype (SASP) factors, such as IL-6 and TNF-α. This induces chronic microenvironmental inflammation, suppresses the activity of muscle stem cells (satellite cells), and drives Fibro-Adipogenic Progenitors (FAPs) towards a fibrotic lineage. This deterioration of the microenvironment, driven by local nuclear senescence, establishes a molecular barrier to re-innervation, ultimately leading to the irreversible progression of sarcopenia (Figure 1b).

##### Dual roles of sensory innervation in regeneration and pathology

The role of sensory nerves within the musculoskeletal system has undergone a radical conceptual evolution. They are no longer viewed merely as passive transmitters of pain, but as active “initiators” and “sculptors” of bone regeneration. However, this innervation possesses a strongly context-dependent role: while indispensable for fracture repair, aberrant nerve ingrowth acts as a central driver of pathology in the IVD, tendon and articular cartilage.

A recent study by Xu et al., utilizing single-cell transcriptomics combined with genetic tracing, revealed that skeletal sensory neurons exhibited a distinct “phase transition” during fracture repair (Xu et al., 2026). In the acute phase (Day 1), neurons predominantly expressed genes associated with nociception (Scn1a) and inflammation (S100a8). However, by the repair phase (Day 14), their transcriptional profile underwent fundamental reprogramming, characterized by the large-scale upregulation of a regenerative gene network including mitogens, angiogenic factors, and osteogenic signals.

Within this complex regenerative interactome, Fibroblast Growth Factor 9 (FGF9) has been identified as a core regulator derived from sensory nerves. It acts directly on FGFR receptors on periosteal mesenchymal stem cells (MSCs) to promote proliferation and osteogenic differentiation. Furthermore, sensory nerves exert exquisite dual control via the secretion of Semaphorin 3A. This molecule not only repels axonal overgrowth (preventing hyperalgesia) but also potently stimulates osteogenic differentiation, ensuring high spatiotemporal synchronization between bone repair and re-innervation (Liu et al., 2023a; Kang and Kumanogoh, 2013). This regulation also involves a local interplay with the sympathetic system, where sensory nerves inhibit local sympathetic tone via the PGE2–EP4 pathway, preventing stem cell adipogenesis and thereby “locking in” an osteogenic fate (Figure 2a) (Hu et al., 2020; Chen et al., 2019).

**FIGURE 2:**
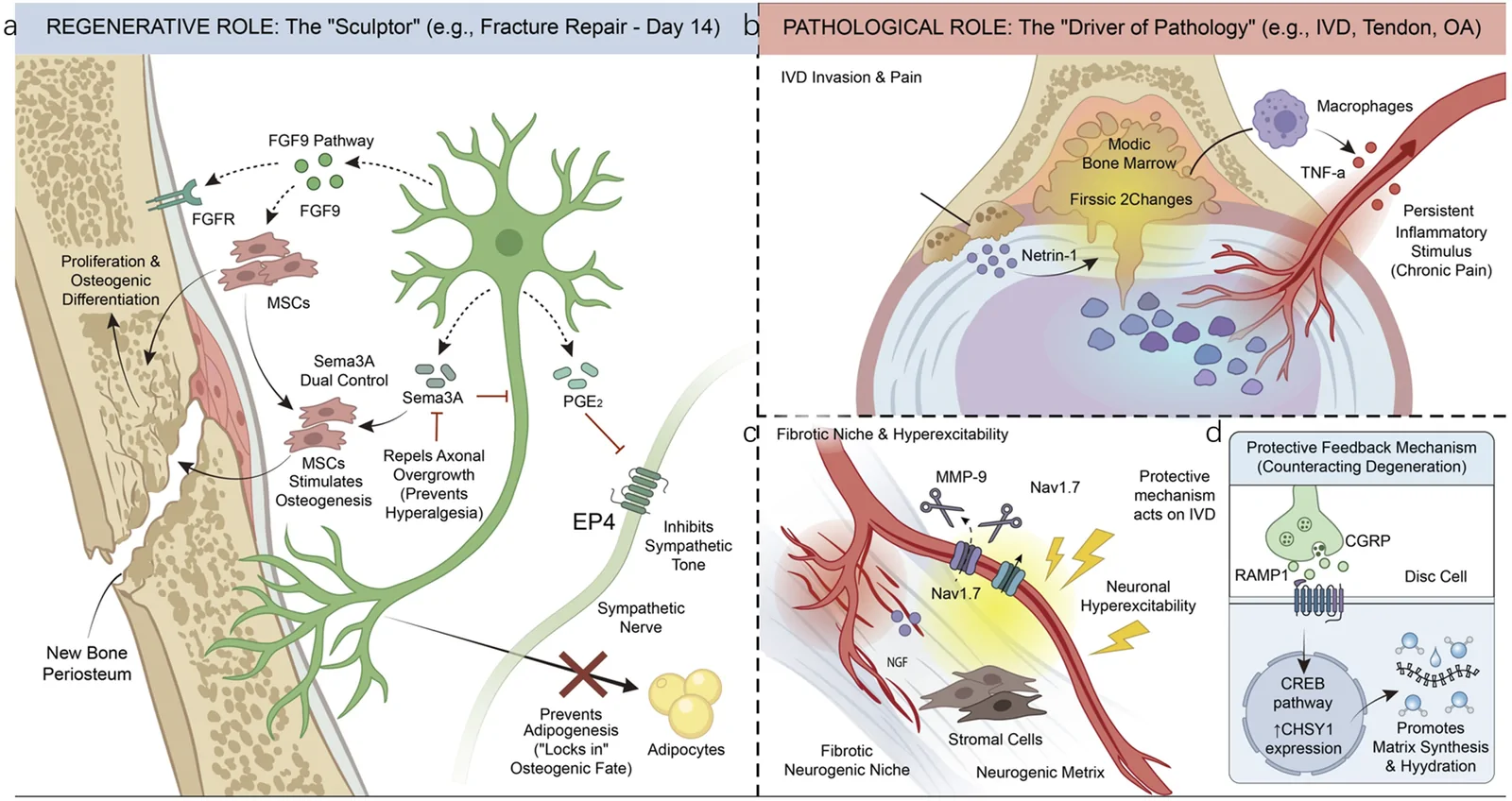
Dual roles of sensory innervation in regeneration and pathology. **(a)** In fracture repair, reprogrammed sensory neurons secrete FGF9 to activate FGFR on periosteal MSCs, while Sema3A promotes osteogenesis yet restrains axonal overgrowth; PGE2–EP4 dampens local sympathetic tone to prevent MSC adipogenesis. **(b)** In degenerate IVD, endplate injury enables invasion guided by osteoclast-derived Netrin-1, and hypoxic NP-derived NGF/BDNF with Modic macrophage TNF-α sustains chronic pain. **(c)** In tendinopathy, a fibrotic neurogenic niche forms as neuronal NGF drives stromal cells toward pro-fibrotic states and hyperexcitability. **(d)** CGRP–RAMP1–CREB induces CHSY1 to support matrix hydration as protective feedback. Abbreviations: BDNF brain-derived neurotrophic factor, CGRP calcitonin gene-related peptide, CHSY1 chondroitin sulfate synthase 1, COX-2 cyclooxygenase-2, CREB cAMP response element-binding protein, EP4 prostaglandin E receptor 4, FGF9 fibroblast growth factor 9, FGFR fibroblast growth factor receptor, IL-1β interleukin-1 beta, IVD intervertebral disc, MMP-9 matrix metalloproteinase-9, MSC mesenchymal stromal/stem cell, Nav1.7 voltage-gated sodium channel 1.7, NGF nerve growth factor, PGE2 prostaglandin E2, RAMP1 receptor activity-modifying protein 1, Sema3A semaphorin 3A, TNF-α tumor necrosis factor-alpha.

In stark contrast to the bone, the healthy IVD is an immune-privileged and aneural structure. Pathological neoinnervation is not only the root source of discogenic low back pain but also represents the breakdown of tissue homeostasis. This process is driven by multiple molecular events, with endplate injury serving as the portal for neural invasion. Unexpectedly, osteoclasts play a key role here by secreting the axon guidance factor Netrin-1, actively guiding sensory nerves through the damaged endplate into the disc interior (Ni et al., 2019). Simultaneously, the hypoxic environment of the degenerate nucleus pulposus triggers massive secretion of NGF and BDNF. This creates a potent neurotrophic gradient that draws nociceptors deep into the nucleus. Clinically common “Modic changes” (vertebral bone marrow signal abnormalities) are, in essence, active biological lesions. Here, macrophages expressing high levels of TNF-α maintain close contact with proliferating nerve fibers, creating a persistent inflammatory stimulus that forms the anatomical substrate of chronic low back pain (Figure 2b) (Ząbek et al., 2025).

Similarly, innervation exhibits a destructive facet in tendinopathy and osteoarthritis (OA). Li et al. proposed the concept of “Fibrotic Neurogenic Niche”, demonstrating that neurons in injured tendons secrete NGF to drive mesenchymal stromal cells towards a pro-fibrotic subpopulation (Li et al., 2025b). In OA, sensory nerves even breach the tidemark to invade the deep cartilage, establishing a malignant crosstalk with senescent synovial fibroblasts (involving RCAN1 and IL-1α). This transforms simple mechanical wear-and-tear into pan-articular neuro-immune inflammation (Figure 2c) (Huo et al., 2026).

However, nerve ingrowth is not entirely devoid of benefit. Recent research has uncovered an often-overlooked protective feedback mechanism: the neuropeptide CGRP, which is released by sensory terminals, acts on the RAMP1 receptor on disc cells to activate the CREB signaling pathway (Hu et al., 2022). This subsequently upregulates the expression of the chondroitin synthase *CHSY1*. This mechanism is crucial for maintaining extracellular matrix hydration, suggesting that the nervous system attempts to counteract disc degeneration by promoting matrix synthesis (Figure 2d).

### Functional crosstalk: neural circuitry and sensorimotor loops

While the molecular crosstalk described above elucidates homeostasis at the tissue level, the most essential function of the nervous system in electrophysiological signal transmission remains the irreplaceable “commander” of musculoskeletal health. In conditions such as SCI, stroke, or peripheral nerve injury (PNI), the pathological origin lies not in metabolic defects of the target organs, but in the physical cessation of neural command transmission. This “electrophysiological silence” triggers a catastrophic cascade, manifesting as functional MSDs (Debenham et al., 2024; Azzollini et al., 2021).

In these functional disorders, the “hardware” of the musculoskeletal system (the effectors) often remains initially intact (Sartori et al., 2021). However, the blockade of upper motor neuron (UMN) or lower motor neuron (LMN) pathways precludes the conversion of action potentials into mechanical contraction at the NMJ. This “command deficit” results not only in the immediate loss of motor function (paralysis) but also dismantles the critical “dual regulation” mechanism between nerve and muscle.

First, “anterograde failure” occurs: deprived of tonic or phasic stimulation, the muscle rapidly enters a state of disuse. This silencing signal activates the intracellular ubiquitin–proteasome pathway, driving the rapid degradation of myofibrillar proteins and subsequent atrophy. Closely following this is “retrograde blockade”. As previously noted, muscle activity itself drives the retrograde transport of neurotrophic factors. Paralysis induced by electrical silence severs the backflow of BDNF and GDNF, leaving surviving neurons to undergo accelerated degeneration due to a lack of “trophic feedback”, thus establishing a vicious cycle (Wang et al., 2016; Tosolini et al., 2022).

The consequences of interrupted electrical signaling extend well beyond the musculature. Without neural-driven muscle contraction, the skeleton is deprived of its primary source of endogenous mechanical loading. In accordance with Wolff’s Law, this “mechanical unloading” rapidly shifts the equilibrium of bone remodeling: osteoblast activity is suppressed, while osteoclasts are hyperactivated, resulting in severe “disuse osteoporosis”, the rate and magnitude of which often far exceed those of postmenopausal bone loss. Consequently, patients suffer a “double blow” comprising both functional loss and structural degeneration.

### Translating biological crosstalk into therapeutic strategies

Taken together, recent research advances have delineated a grand physiological landscape: the nervous system, skeleton, and musculature are no longer viewed as anatomically distinct entities, but rather as a “community of shared biological fate”, intricately interwoven via synaptic connections, paracrine factors, and systemic hormones. This paradigm shift fundamentally overturns our understanding of the locomotor system: it functions not merely as a mechanical scaffold for the body, but as a critical endocrine hub regulating systemic metabolism and cognitive function. Within this complex interaction network, the flow of information is both omnidirectional and dynamic.

Predicated on these pathological mechanisms, the application of NIs in MSDs is no longer confined to the role of simple assistive technologies. Instead, NIs are poised to evolve into true therapeutic interventions capable of reversing tissue pathology. Such artificially induced electrical activity exerts profound biological effects: beyond simply eliciting muscle contraction to restore limb movement (function), it modulates homeostasis from the perspective of musculoskeletal interoception, thereby promoting tissue repair and regeneration. Consequently, future NI technologies essentially target the “neuro-musculoskeletal axis”, aiming to reverse downstream metabolic decline by re-establishing normal electrophysiological activity.

## Recent applications of neural interfaces in musculoskeletal disorders

### Applications in structural musculoskeletal disorders

Over the past 5 years, the therapeutic paradigm for structural MSDs has undergone a profound transformation. Driven by the deep integration of neural interface technologies, advanced biomaterials, and digital modelling, clinical interventions are rapidly evolving from traditional passive mechanical support and palliative pharmacotherapy towards active, closed-loop neurobiological modulation.

In this section, we focus on four key domains: spinal disorders, articular pathologies, soft tissue injuries, and metabolic bone diseases. We systematically analyze how NIs are achieving the leap from mere symptom masking to homeostatic restoration by reshaping neuro-tissue interactions.

#### Applications in spinal disorders

Chronic low back pain (CLBP) and IVD degeneration constitute one of the highest burdens of disability globally. Given the complex cellular and molecular pathology of IVDD—including nucleus pulposus apoptosis, matrix degradation, and aberrant neurovascular ingrowth—alongside the maladaptive central remodeling caused by CLBP (Nie et al., 2025; Deng et al., 2023; Fidai et al., 2025; Guarnaccia et al., 2025; Zou et al., 2026; Green et al., 2025), traditional pharmacological and surgical approaches often prove inadequate. Against this backdrop, bio-electronic systems are redefining the boundaries of spinal care. While NIs have a long history in the spine, technological iterations over the last 5 years have ushered in a new era of “precision modulation”. Traditional open-loop stimulation is being rapidly superseded by closed-loop systems with physiological feedback, whilst the introduction of neuromorphic devices is elevating intervention to the level of molecular modification.

##### Closed-Loop SCS and ECAP Feedback

Spinal Cord Stimulation (SCS) remains a gold standard for refractory neuropathic pain (de Vos and Meier, 2024). However, traditional open-loop SCS suffers from a fundamental physical limitation: physiological movements such as respiration or postural changes alter the distance between the spinal cord and the electrodes, causing drastic fluctuations in current density (Shirvalkar and Poree, 2023). To address this, closed-loop SCS systems based on Evoked Compound Action Potentials (ECAPs) (Mangano et al., 2025)—such as the Inceptiv (Mohabbati et al., 2025) and Evoke (Mekhail et al., 2020) systems—have achieved breakthrough progress.

ECAPs directly reflect the volume of activated nerve fibers. Using microsecond-level real-time feedback algorithms, these systems compare measured ECAP amplitudes against a preset target, automatically adjusting current intensity. Clinical data from the SOLIS (North et al., 2024; North et al., 2025) and Evoke trials confirm that this mechanism not only eliminates the discomfort of over-stimulation but also optimizes GABA release in the dorsal horn via sustained, stable Aβ fiber activation. This significantly improves quality of life and reduces opioid dependence in patients with refractory pain (Mangano et al., 2025; Parker et al., 2020). To provide a clearer, side-by-side view of recent progress in ECAP-guided closed-loop SCS, Table 1 summarizes the key systems and representative clinical studies (Anitescu et al., 2025; Latif et al., 2025).

**TABLE 1:** Clinical applications in Closed-Loop SCS and ECAP Feedback for CLB**P**.

Name	Feedback signal source	Adjustment frequency	Clinical evidence in low back pain	Battery technology	MRI compatibility	Programming automation	FDA approval	Reference
Saluda medical (Evoke®)	Spinal cord evoked compound action potential (ECAP)	Pulse-by-pulse adjustment; thousands of updates per second; real-time response to microsecond-level changes	EVOKE RCT (36 months): • Responder rate 78% • 0% explant due to loss of efficacy • Superior to open-loop systems	Rechargeable (designed longevity >10 years)	Conditional: • 1.5T/3T head/limbs • 1.5T full body • Limited when lead fracture occurs	EVA™ platform (2025): • Auto-generates closed-loop program within 13 min	yes	(Mekhail et al., 2020)
Medtronic (Inceptiv™)	Spinal cord evoked compound action potential (ECAP)	50 Hz sampling adjustment; 50 times/s (3000/min), sufficient to handle postural changes	ECHO-MAC (12 months): • Responder rate 82% • 93% reduction in overstimulation • 90% of patients preferred closed loop	Overdrive™: • Fully charged in 1 h • 15-year longevity (95% capacity)	Industry-leading: • 1.5T and 3T full-body unrestricted • Impedance independent (scannable even with lead fracture/high impedance)	DTM™ integration: • combines DTM waveform optimization with closed-loop control	yes	(Mohabbati et al., 2025)
Boston Scientific (WaveWriter Alpha™)	Preset algorithms and duty cycle	Periodic adjustment according to a schedule	SOLIS/Real-world: • Rapid onset (within minutes) • High responder rate; suitable for refractory back pain	Rechargeable (Gen 1) and non-rechargeable (Prime)	ImageReady™: • 1.5T full body • Requires impedance limit (<15 kΩ)	Illumina 3D: • Anatomy-based electric-field navigation	yes	(Anitescu et al., 2025)
Nevro (HFX iQ™)	Patient app input + cloud-based AI data	Not real-time; recommendations based on daily/weekly feedback	SENZA-RCT/NSRBP: • Long-term evidence for 10 kHz high-frequency therapy • Suitable for non-surgical back pain patients	Rechargeable (10-year longevity)	Conditional: • 1.5T/3T full body • Must meet impedance limits	AI recommendations: • Parameter recommendations based on similar patient cohorts	yes	(Latif et al., 2025)

Abbreviations: AI, artificial intelligence, CLBP, chronic low back pain, DTM, differential target multiplexed (therapy), ECAP, evoked compound action potential, FDA (U.S.) food and drug administration, Hz Hz, kHz kHz, kΩ kilo-ohm, MRI, magnetic resonance imaging, NIs, neural interfaces, RCT, randomized controlled trial, SCS, spinal cord stimulation, T T (e.g., 1.5T/3T = 1.5-/3-tesla).

##### Neuromorphic interfaces and regenerative modulation

While closed-loop SCS solves stability issues, its primary goal remains pain blockade. Recently, a “Neuromorphic Peripheral Somatosensory–Computer Interface (PSCI)” based on atomically thin molybdenum disulfide (MoS2) transistor arrays has introduced a revolutionary perspective on spinal regeneration (Wang et al., 2025a). Compared to traditional silicon-based electronics, these 2D materials boast an on/off ratio as high as 1.31 \times 10^7^ and an ultra-low subthreshold swing (0.07 V/dec), enabling the capture of faint bioelectric signals in the dorsal root ganglion (DRG) with varying sensitivity.

Critically, research has identified a specific neural frequency band (0–20 Hz)—termed the “regenerative band”, which was found to be highly correlated with the release of the key neurotrophic factor of calcitonin gene-related peptide (CGRP). A closed-loop system built on this discovery moves beyond simple blocking currents; it monitors the neural state in real-time and delivers specific “Regenerative Waves (R-waves)” on demand. This biomimetic stimulation strategy has successfully upregulated CGRP expression in animal models, suppressing inflammatory factors (COX2, IL-1β) whilst significantly promoting cartilage matrix synthesis (Aggrecan, Col2a1). This marks the transition of spinal bioelectronic medicine from symptom control to homeostatic regulation (Figure 3).

**FIGURE 3:**
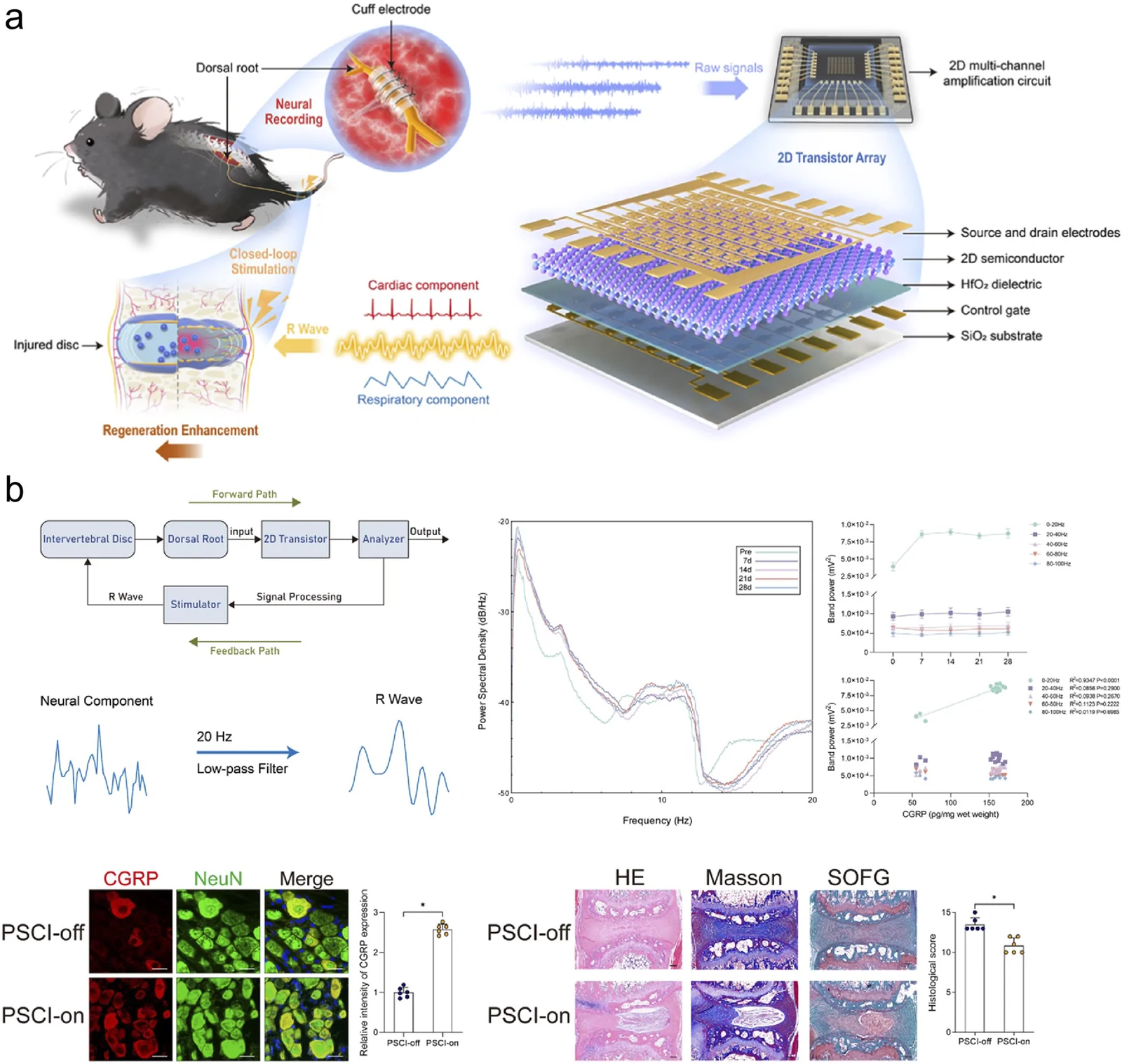
PSCI for IVD regeneration. **(a)** Schematic of the PSCI system integrating neural recording and targeted electrostimulation for regenerative modulation. A cuff electrode encircles the dorsal root innervating the disc to capture interoceptive signals, which are processed by a 2D multi-channel amplification array (2D-MCAA) to extract the regeneration waveform (R wave). **(b)** Real-time R wave–based feedback stimulation maintains consistent regenerative signal power within the 0–20 Hz range, which is correlated with increased CGRP expression in PSCI-on group. Immunofluorescence of CGRP and NeuN in L6 DRG and histological staining (HE, Masson, SOFG) of Co4–5 discs reveals enhanced extracellular matrix organization and tissue repair under PSCI activation (P < 0.05, n = 6). Abbreviations: 2D two-dimensional, CGRP calcitonin gene-related peptide, HE hematoxylin and eosin (staining), HfO2 hafnium dioxide, Hz Hz, IVD intervertebral disc, Masson Masson’s trichrome (staining), NeuN neuronal nuclei (RBFOX3; neuronal marker), PSCI (bioelectronic) closed-loop stimulation system (as defined in your manuscript), SiO2 silicon dioxide, SOFG Safranin O–Fast Green (staining).

##### Functional restoration and precision targeting

Although the following strategies rely on neuro-modulatory mechanisms traditionally associated with functional restoration, they are discussed here within the context of structural spinal disorders. This is because their primary therapeutic target is not the replacement of lost neural commands, but the correction of structurally driven neuromuscular dysfunction, specifically, restoring deep spinal stability and biomechanical integrity secondary to chronic mechanical pathology. For the source control of mechanical low back pain, restorative neurostimulation offers a new strategy to break the “pain–inhibition–instability” cycle. By addressing atherogenic muscle inhibition of the multifidus, systems like ReActiv8 induce tetanic contraction of deep stabilizing muscles via direct stimulation of the L2 medial branch nerve. This peripherally driven proprioceptive input aims to reawaken motor control circuits dormant in the brain. Long-term follow-up data have confirmed its cumulative effect in reversing muscle atrophy and restoring spinal stability (Gilligan et al., 2023a; Gilligan et al., 2023b; Schwab, 2024; Gilligan, 2024; Aldabek et al., 2024). For specific pain types such as complex regional pain syndrome, DRG stimulation has emerged as a paradigm of precision neuromodulation, demonstrating superior efficacy to traditional SCS due to its high dermatomal specificity and low energy consumption (Rigoard et al., 2021; Han and Cong, 2024).

##### Bioelectronic systems

Beyond neuromodulation, an increasing number of bio-electronic hybrid systems have been designed to directly intervene in structural failure associated with spinal disorders, such as impaired osseous fusion, delayed fracture healing, and postoperative mechanical instability. Smart spinal fusion cages (e.g., the SmartFuse system) integrate micro-sensors and therapeutic units to actively participate in the bone–implant interface (Murphy, 2024). By electrically upregulating osteoblast activity, these devices not only accelerate bone formation and fusion but also overcome the intrinsic delay of postoperative radiographic evaluation by quantitative assessment of osseointegration and fusion maturity via real-time impedance monitoring (Kim et al., 2022). Moving beyond static implants, piezoelectric smart sensors exploit the piezoelectric effect to convert physiological spinal loading into electrical signals, thereby enabling battery-free, self-powered monitoring of load transmission and construct stability. Such systems provide continuous feedback on mechanical integrity, which is critical for detecting micro-instability or non-union at an early stage. In the context of regenerative medicine, pulsed electromagnetic fields and piezoelectric scaffolds further extend bio-electronic strategies from monitoring to *active structural repair*. These platforms counteract the progressive structural collapse characteristic of IVD degeneration by restoring metabolic homeostasis of nucleus pulposus cells, coupling mechanical cues with electrical stimulation (ES), and activating the SIRT1–autophagy pathway and RhoA/ROCK1 signaling axis (Zheng et al., 2022; Huang et al., 2024).

#### Applications in articular diseases

In the field of joint pathology, the application of NIs presents a dual landscape: systemic immunomodulation for Rheumatoid Arthritis (RA) and local regenerative repair for OA.

##### Rheumatoid arthritis

Bioelectronic medicine has achieved a historic breakthrough in the treatment of the autoimmune disease of RA. Based on the “Inflammatory Reflex” theory, vagus nerve stimulation (VNS) has proven to block pro-inflammatory cytokine release at the molecular level via the splenic nerve-spleen axis by utilizing the interaction between acetylcholine and α7nAChR (Lerman et al., 2025; Peterson et al., 2024). The US Food and Drug Administration (FDA) has approved the SetPoint system as a clinical therapy for RA. The pivotal RESET-RA trial demonstrated that for patients with drug-refractory RA, implantable 5NS significantly improved the ACR20 response rate and offered structural protection against bone erosion with no adverse effect of systemic immunosuppression (Bonaz, 2024; Chowdhury et al., 2025). Furthermore, to enhance the targeting efficacy and reduce cardiovascular adverse effects, direct splenic nerve stimulation and non-invasive focused ultrasound targeting the spleen are developing rapidly (Dong et al., 2025; Kocyigit et al., 2024). The latter offers a non-invasive option for home-based anti-inflammatory therapy by activating splenic ion channels via external mechanical energy. To provide a clearer overview of the rapidly evolving clinical landscape in RA bioelectronic therapy, Table 2 summarizes representative devices and recent studies (Gaylis et al., 2025; Courties et al., 2025; Baker et al., 2023).

**TABLE 2:** Clinical applications in Rheumatoid Arthritis (RA).

Name	Company	Technology type	Target	Invasiveness	Power supply	Clinical/regulatory status (up to 2026)	Product features and key advantages	References
SetPoint system	SetPoint medical	Vagus nerve stimulation (VNS)	Cervical vagus nerve	Implantable (minimally invasive)	Implantable battery (wireless charging)	FDA approved (2025) *(based on the RESET-RA trial)*	First FDA-approved therapy for RA; minimally invasive; “pod-shaped” form factor; iPad-based digital dose control; no need for subcutaneous tunneling.	(Gaylis et al., 2025)
Galvani system	Galvani bioelectronics	Splenic nerve stimulation	Splenic neurovascular bundle	Implantable (laparoscopy)	Implantable battery (wireless charging)	Clinical RCT phase *(FDA approval progressing toward pivotal trials)*	Precise targeting with reduced cardiac/laryngeal side effects; backed by GSK/Verily; potential for closed-loop development.	(Dong et al., 2025)
Parasym	Parasym Ltd	taVNS	Auricular branch of the vagus nerve	Non-invasive (wearable)	External battery	Clinical research phase *(NCT06741891)*	Non-invasive and low cost; may reduce CRP/IL-6; effect depends on daily adherence/wearing for maintenance.	(Courties et al., 2025)
Sparrow	Spark biomedical	tAN	Auricular nerve branches	Non-invasive (wearable)	External battery	Clinical research phase *(exploring RA indications)*	Multimodal transcutaneous stimulation; primarily approved for smoking cessation, now expanding to inflammation/autoimmune indications.	(Baker et al., 2023)

CRP C-reactive protein, GSK GlaxoSmithKline, IL-6 interleukin-6, NCT National Clinical Trial (ClinicalTrials.gov identifier), NIs, neural interfaces, RA rheumatoid arthritis, RCT randomized controlled trial, taVNS transcutaneous auricular vagus nerve stimulation, tAN transcutaneous auricular nerve (stimulation), VNS vagus nerve stimulation.

##### Osteoarthritis

In contrast to the systemic intervention for RA, OA treatment focuses on long-term local analgesia and tissue regeneration. In the realm of Peripheral Nerve Stimulation (PNS), short-term percutaneous systems (e.g., Sprint) bridge the gap between pharmacotherapy and arthroplasty by utilizing neural plasticity to induce sustained pain relief via a 60-day temporary implant (Chitneni et al., 2021; Hasoon et al., 2025). This is particularly applicable for chronic post-TKA pain management.

By addressing the fundamental pathology of OA-cartilage defects, regenerative bioelectronics shows remarkable promise (Liu et al., 2025a; Huang et al., 2018; Wu et al., 2025a). Novel injectable piezoelectric hydrogels ingeniously harness the mechanical forces of joint movement by converting them into micro-electric fields via the piezoelectric effect. These endogenous electrical signals activate voltage-gated calcium channels on chondrocytes, thus triggering the Calcineurin–NFAT signaling pathway and subsequently upregulating growth factors such as TGF-β1. Animal studies have demonstrated that this “battery-free” strategy induces regeneration of the high-quality hyaline cartilage, representing a leap from symptom control to disease-modifying osteoarthritis drugs. To enable a direct comparison of these emerging OA bioelectronic strategies, Table 3 summarizes representative PNS platforms and regenerative bioelectronic approaches (Kalia et al., 2022; Luna et al., 2025; Lin et al., 2025).

**TABLE 3:** Clinical applications in Osteoarthritis (OA).

Name	Company	Technology type	Target	Invasiveness	Power supply	Clinical/regulatory status (up to 2026)	Product features and key advantages	References
SPRINT PNS	SPR therapeutics	Temporary peripheral nerve stimulation (PNS)	Femoral nerve/sciatic nerve/peroneal nerve	Percutaneous stimulation (removed after 60 days)	External pulse generator (temporary implant)	FDA approved and marketed; supported by extensive clinical data	60-day therapy with sustained long-term relief; no permanent implant; micro-lead technology; may induce central neuroplasticity.	(Chitneni et al., 2021)
Nalu system	Nalu medical	Miniaturized implantable PNS	Peripheral nerves (e.g., nerves around the knee)	Implantable (ultra-miniature)	No implanted battery (externally RF-powered)	FDA approved and marketed; widely used	Smallest volume (∼1.5 cc); minimally invasive implantation; long service life (no battery degradation); suitable for joint locations.	(Kalia et al., 2022)
StimRouter	Bioventus	Implantable PNS	Peripheral nerves (e.g., around the knee)	Implantable	Powered by an external transmitter	FDA approved and marketed	Long-term implant solution targeting a single nerve; substantial clinical data accumulated for knee OA.	(Luna et al., 2025)
Piezoelectric Gel	UConn (Nguyen lab)	Regenerative bioelectronic scaffold	Articular cartilage	Injectable (intra-articular)	Self-powered (electricity generated by motion/ultrasound)	Preclinical/large-animal stage *(NIH funding through 2029)*	Regenerative medicine breakthrough; promotes cartilage regeneration via bioelectric signals; drug-free and self-powered.	(Lin et al., 2025)

FDA (U.S.) food and drug administration, NIH (U.S.) national institutes of health, NIs, neural interfaces, OA osteoarthritis, PNS, peripheral nerve stimulation, RF, radiofrequency, UConn, University of Connecticut.

#### Applications in soft tissue diseases

Muscle and ligament repair has long faced the clinical dilemma of asynchronous “structural continuity restoration” and “functional integration”. Particularly in volumetric muscle loss (VML), simple biomaterial fillers often fail to reconstruct complex neuromuscular junctions (NMJs) and vascular networks. Research over the past 5 years indicates that future solutions lie in the deep fusion of precise structural biomimicry with active electrophysiological modulation.

##### Muscle repair and regeneration

In VML repair, tissue engineering is undergoing a paradigm shift from “passive structural substitution” to “active functional reconstruction”. The newly developed “Electrical Functional Neuro-vascularized Engineered Muscle (EFNEM)” overcomes the ceiling of traditional scaffold regeneration through a dual-regulation strategy (Ding et al., 2026). First, it provides critical physical guidance for myocyte alignment and fusion by utilizing light-induced anisotropic cell sheet technology to mimic topology of the natural muscle basement membrane. This highly ordered microstructure not only enhances the efficiency of force transmission but also constructs a “highway” for parallel ingrowth of vessels and nerves. However, structural modification alone is insufficient. To activate the “engine” of regeneration, the system integrates high-resolution 3D-printed flexible electrodes. These electrodes, exhibiting excellent tissue compliance, deliver precise ES *in vivo* to simulate the endogenous bioelectric environment. Studies have demonstrated that the synergy of structural alignment and ES produces a significant multiplier effect, whereby electrical signals not only promote myofiber maturation and hypertrophy but also accelerate NMJ formation and functional vascularization. This “Photo-Electro-Bio” combined strategy offers a highly translational paradigm for resolving the bottleneck of delayed neovascularization in VML (Figure 4).

**FIGURE 4:**
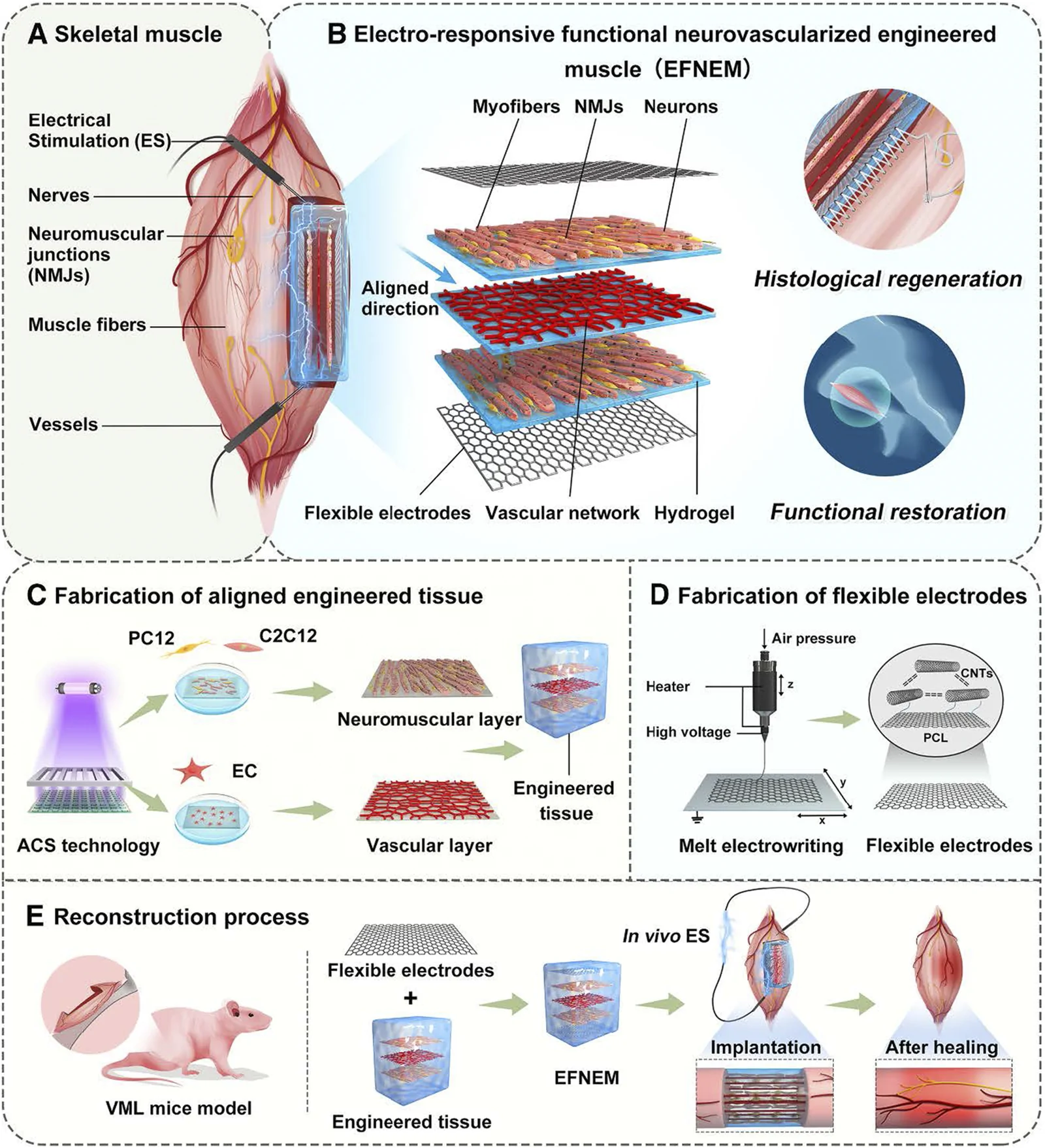
Electrical functional neuro-vascularized engineered muscle (EFNEM) for volumetric muscle loss (VML) repair. **(A)** Native skeletal muscle architecture highlighting nerves, NMJs, vessels and the application of ES. **(B)** EFNEM integrates aligned myofibers, neuro-muscular components and a vascular network within a hydrogel scaffold, coupled with flexible electrodes to enable electro-responsive regeneration and functional restoration. **(C)** ACS technology guides myocyte alignment/fusion and provides topological cues for parallel neurovascular ingrowth. **(D)** Delivery of precise ES *in vivo* by fabricating high-compliance and high-resolution flexible electrodes. **(E)** Reconstruction workflow in a VML mouse model: assembly, implantation, ES, and post-healing recovery. Abbreviations: ACS acoustic cell/structure (alignment) technology, C2C12 mouse myoblast cell line (C2C12), CNTs carbon nanotubes, EC endothelial cells, EFNEM electro-responsive functional neurovascularized engineered muscle, ES electrical stimulation, NMJs neuromuscular junctions, PC12 rat pheochromocytoma cell line (PC12), PCL poly(ε-caprolactone), VML volumetric muscle loss.

Unlike permanent implants, transient electronics have demonstrated unique advantages for acute muscle injury or nerve repair. Implantable stimulators based on biodegradable metals like molybdenum and magnesium can provide precise ES during the critical therapeutic window. By activating the PI3K/Akt/mTOR pathway, they promote protein synthesis and inhibit atrophy, before degrading completely within the body, thus avoiding the risks associated with secondary removal surgeries (Ahn et al., 2025). More advanced closed-loop neuromuscular modulation systems integrate multimodal sensors to perceive healing stages in real-time via tissue impedance changes, adaptively adjusting stimulation parameters for personalized rehabilitation. The application of conductive hydrogels further resolves the mechanical mismatch between rigid electronics and soft tissue, serving both as signal-transmitting electrodes and as cell scaffolds to promote myoblast electrical coupling (Bi et al., 2025).

##### Ligament and Tendon Repair

In ligament and tendon repair, the research focus has returned to the tissue natural property of piezoelectricity. While traditional scaffolds often lack a physiological electrical microenvironment, novel piezoelectric bio scaffolds (e.g., PVDF-TrFE nanofibers) mimic the piezoelectric effect of collagen fibers (Fernandez-Yague et al., 2021). Upon mechanical stretching, the scaffold generates micro-potentials that specifically activate mechanosensitive ion channels like Piezo2. Through mechano-electrical transduction, this promotes stem cell differentiation towards tenogenic lineages and guides the ordered arrangement of collagen fibers, thus significantly enhancing the mechanical strength of the neo-tissue. Additionally, smart implants integrated with flexible strain sensors are entering the clinical horizon. By monitoring graft tension in real-time and wireless warning of overload risks, they provide a quantitative basis for precise rehabilitation following anterior cruciate ligament reconstruction, marking the advance of orthopedic implants towards digitalization and intelligence (Kelmers et al., 2023). Concurrently, VNS accelerates inflammation resolution and reduces fibrosis by promoting the synthesis of specialized pro-resolving mediators, offering a systemic immune-optimized environment for soft tissue healing (Caravaca et al., 2022).

#### Applications in skeletal and bone metabolic disorders

The rise of orthopedic bioelectronic medicine is founded on a renewed understanding of skeletal interoception, where the bone is redefined as an endocrine organ under the fine control of the nervous system.

##### Osteoporosis and Metabolic Bone Disease

In osteoporosis, VNS demonstrates potential to intervene in bone metabolism by regulating autonomic balance. Pilot clinical studies have shown that VNS inhibits osteoclast activation via the cholinergic anti-inflammatory pathway, which significantly increases bone density (Tamimi et al., 2021; Courties et al., 2021). To reduce invasiveness, minimally invasive magnetic VNS (mVNS) (Figure 5) (Wang et al., 2025b) and ES targeting the DRG are under development; the latter directly stimulates osteogenesis and improves bone microcirculation by promoting CGRP release (Wang et al., 2023). In metabolic bone diseases such as osteomalacia, the combination of implantable ion sensors and closed-loop drug delivery systems enables real-time monitoring and precise regulation of calcium, phosphorus, and vitamin D metabolites, providing powerful technical support for recurrence warning and treatment in complex conditions like tumor-induced osteomalacia (TIO) (Chande and Bergwitz, 2018; Rani et al., 2020; Dahir et al., 2021).

**FIGURE 5:**
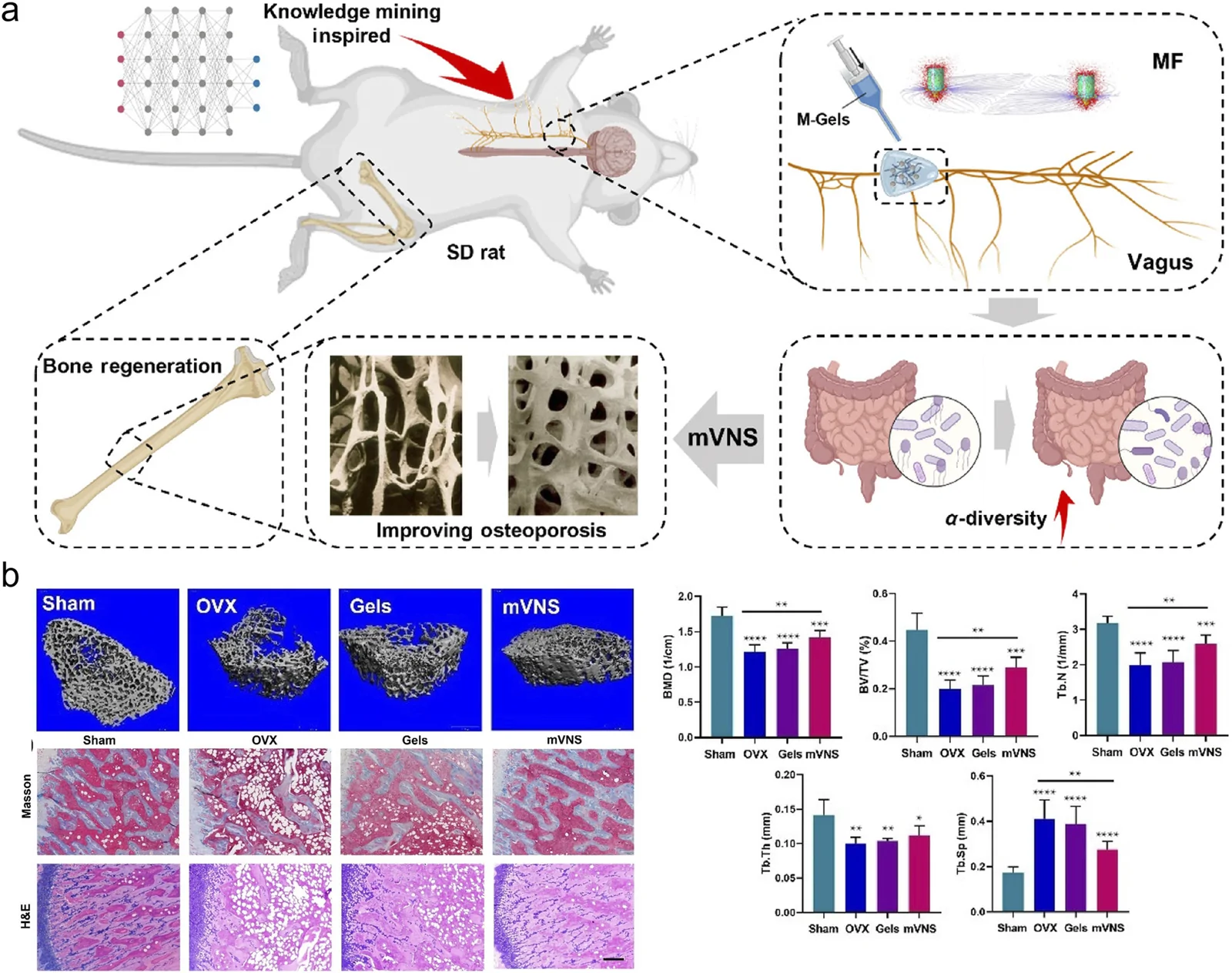
Knowledge-mining–guided mVNS alleviates ovariectomy–induced osteoporosis. **(a)** Schematic of the mVNS concept: magnetic gels (M-Gels) placed around the cervical vagus nerve enable stimulation under a mild rotational magnetic field, with proposed downstream benefits on bone regeneration and gut microbiota (α-diversity). **(b)** Representative 3D micro-CT reconstructions and femoral histology (Masson, H&E) from Sham, OVX, Gels and mVNS groups after 16-week treatment. Quantification of bone micro-CT indices (BMD, BV/TV, Tb.N, Tb.Th, Tb.Sp). Data are mean ± s.d. (n = 5); *P < 0.05, **P < 0.01, ***P < 0.005, ****P < 0.001. Abbreviations: BMD bone mineral density, BV/TV bone volume/total volume, H&E hematoxylin and eosin (staining), MF magnetic field, mVNS microgel-mediated vagus nerve stimulation, OVX ovariectomy/ovariectom.

##### Fracture and non-union

In the management of fractures and non-unions, smart bone plates integrated with micro-strain gauges can record force transmission curves during healing. This allows for the precise diagnosis of non-union and guides weight-bearing rehabilitation, surpassing the limitations of traditional imaging (Schulz et al., 2025; Ledet et al., 2018). Transient electronics also hold immense potential here; released magnesium and zinc ions not only serve as electrode materials but possess intrinsic osteogenic bioactivity (Wang et al., 2025c; Hosseini et al., 2020). For refractory lesions such as diabetic fractures, bioelectronic coatings that release active ions in response to high-glucose environments offer a novel solution for improving the local microenvironment (SanVicente et al., 2023).

In summary, application of NIs in structural MSDs has expanded from singular symptom control to the deep modulation of tissue regeneration, immune microenvironments, and systemic metabolism. From decoding the “electrophysiological codes” of spinal regeneration using 2D MoS2 neuromorphic interfaces to achieving active functional reconstruction of VML via EFNEM, these breakthroughs collectively point to a clear future: orthopedic treatment is evolving into an intelligent ecosystem where mechanical support, biomaterials, and electronic information are seamlessly fused. By constructing bio-electronic closed loops capable of sensing, responding, and self-regulating, we are gradually realizing the precise reversal of pathological processes and the complete reshaping of tissue function.

Taken together, neural-interface strategies for structural musculoskeletal disorders differ substantially in therapeutic objective, clinical maturity, and translational burden. SCS, DRG stimulation, VNS, and PNS have comparatively established clinical pathways and well-defined neuromodulatory endpoints, but evidence that they directly modify underlying tissue degeneration remains condition-dependent. In contrast, piezoelectric scaffolds, bioelectronic coatings, and other regenerative interfaces can couple mechanical or biochemical cues to local tissue repair, yet their supporting evidence remains predominantly preclinical. Thus, current clinical maturity favors conventional neuromodulation, whereas regenerative bioelectronics offers broader disease-modifying potential but faces greater uncertainty regarding durability, power supply, manufacturing, and long-term biocompatibility.

### Applications of neural interfaces in functional musculoskeletal disorders

Functional MSDs stem from abnormalities in the neural signals that innervate the musculoskeletal system. Consequently, current therapeutic strategies are shifting from passive rehabilitation to active reconstruction based on NIs. In this section, we review three key advancements: brain-spinal interfaces, closed-loop modulation, and regenerative bioelectronics. By analyzing mechanisms such as ECAP feedback, Hebbian plasticity, and biological amplification, we elucidate how intelligent bioelectronic systems integrate signal recording and stimulation with advanced algorithms to achieve precise functional restoration of the neuromusculoskeletal axis.

#### Applications in CNS injuries

CNS injuries, whether stemming from physical spinal cord transection or cortical network disruption due to cerebrovascular accidents—fundamentally result in the functional decoupling of descending motor commands from downstream execution networks. While traditional biological repair strategies are constrained by the limited intrinsic regenerative capacity of the CNS, emerging neural interface technologies offer an alternative approach. By utilizing engineering methods to establish artificial communication links or modulate the excitability of residual circuits, these technologies provide a novel dimension for functional recovery.

##### Spinal cord injury

In the management of autonomic function, closed-loop systems demonstrate unparalleled advantages. Taking hemodynamic stability as an example, patients with high-level SCI often suffer from orthostatic hypotension (OH) due to the loss of sympathetic control. The recently developed ARC-IM system utilizes an implanted blood pressure sensor to monitor arterial pressure fluctuations in real time. By combining this with posture changes detected by an accelerometer, it establishes an artificial “electronic baroreflex” loop (Engel-Haber et al., 2024). Upon detecting the onset of hypotension, the system automatically activates stimulation targeting the T11-L2 spinal segments. This precisely excites the sympathetic preganglionic neurons innervating the splanchnic major vascular bed, inducing abdominal vasoconstriction to maintain cerebral perfusion. Clinical evidence indicates that this closed-loop intervention not only instantaneously corrects blood pressure abnormalities but also leads to permanent improvements in basal vascular tone through long-term neuroplasticity mechanisms, thereby liberating some patients from reliance on vasopressors (Phillips et al., 2025; Soriano et al., 2025; Trivano, 2025).

Similarly, closed-loop control targeting the neurogenic bladder addresses the persistent challenges of incontinence and renal damage in SCI patients (Raczkowska et al., 2019; Vogel and Nakib, 2025). Utilizing signals from the dorsal root ganglion or bladder wall impedance sensing technology, these systems can intelligently distinguish between the storage and voiding phases. During the storage phase, the system employs high-frequency inhibitory stimulation to block spasmodic contractions of the detrusor muscle; conversely, during the voiding phase, it delivers low-frequency burst stimulation to synchronously activate the detrusor muscle and relax the urethral sphincter. Animal models and early clinical data demonstrated that this synergistic control strategy increased voiding efficiency from 40% under open-loop modes to over 70%, significantly reducing residual urine volume and the risk of urinary tract infections.

If SCS activates dormant spinal circuits (Wagner et al., 2018; Rowald et al., 2022; Kathe et al., 2022), the Brain-Spine Interface (BSI) endows these circuits with the capacity for conscious control (Lorach et al., 2023). A landmark study by Courtine et al., in 2023 demonstrated the successful application of the world’s first fully implanted BSI system in a patient with complete SCI, a concept vividly termed the “Digital Bridge.” The hardware architecture of the system comprises two core components: the WIMAGINE® recorder located epidurally over the motor cortex and the ARC-IM® stimulator located epidurally over the lumbosacral spinal cord. Its operational workflow involves not merely signal transmission but a complex process of decoding and re-encoding. When the patient generates a motor intention to walk, high-dimensional neural signals captured by the cortical electrodes are decoded in real-time by deep learning algorithms within a portable processing unit. These signals are translated into specific motor commands (e.g., “hip flexion” or “knee extension”) and subsequently transmitted wirelessly to the spinal stimulator. Based on these commands, the stimulator delivers spatiotemporally precise sequences of electrical pulses to specific dorsal roots, activating the motor neuron pools responsible for the corresponding muscle groups (Figure 6).

**FIGURE 6:**
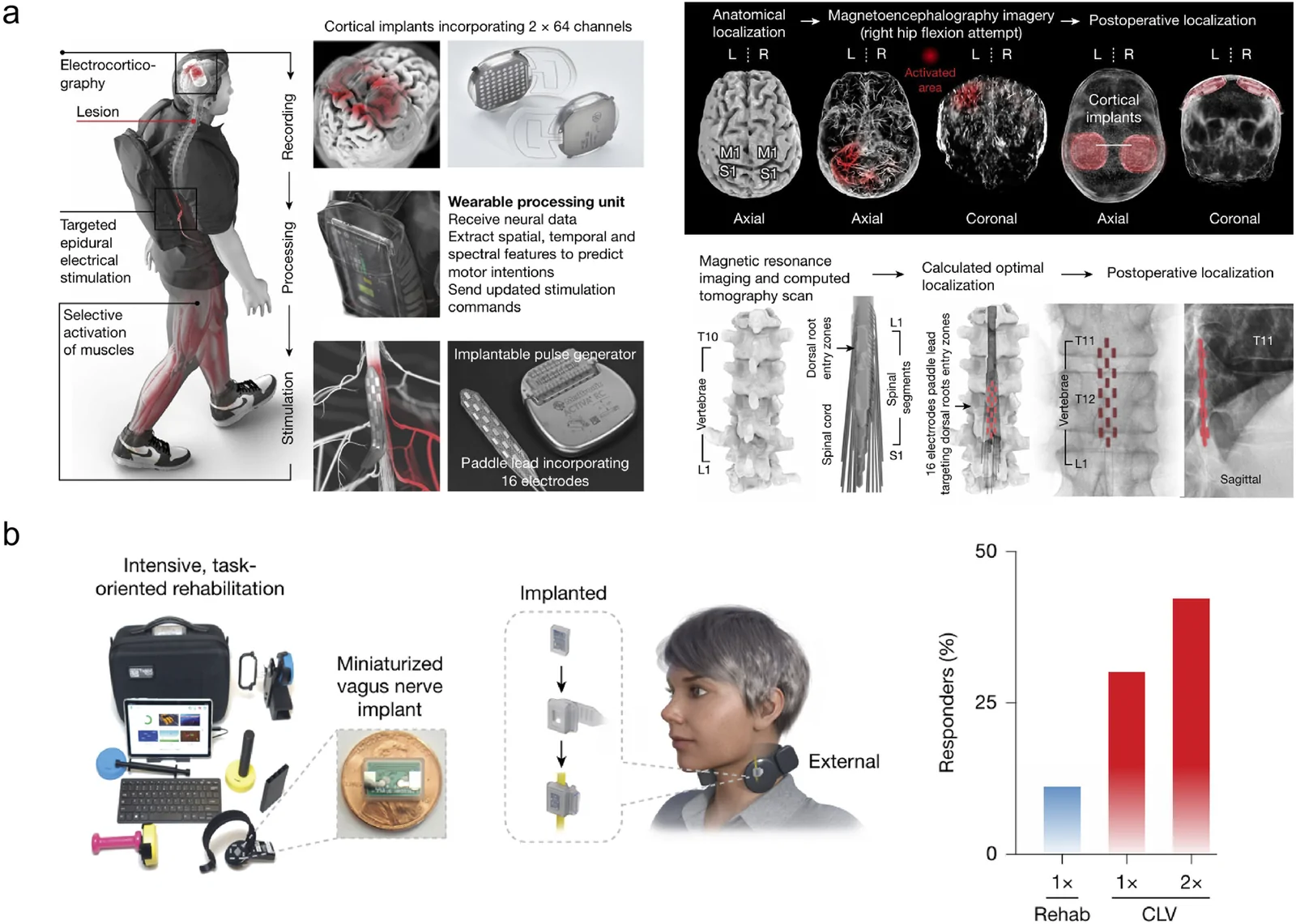
Closed-loop neural interface for motor recovery after spinal cord injury. **(a)** Design and implantation of a BSI. Two 64-channel cortical implants are positioned epidurally over the sensorimotor cortex to record ECoG signals, which are decoded by a processing unit to predict motor intentions. These predictions dynamically modulate epidural stimulation programs delivered to the dorsal root entry zones of the lumbosacral spinal cord via an implantable pulse generator and a 16-electrode paddle lead, enabling volitional control of movement. **(b)** CLV integrates intensive, task-specific rehabilitation with real-time, sensor-guided neuromodulation. A miniaturized implanted stimulator, powered by an external controller, delivers phase-locked VNS during successful movements, promoting adaptive neuroplasticity and driving superior motor recovery compared with conventional training. Abbreviations: BSI, brain–spine interface; ECoG, electrocorticography; CLV, closed-loop vagus nerve stimulation; VNS, vagus nerve stimulation.

This millisecond-level synchronization of “cortical intention-spinal activation” not only achieves immediate control over paralyzed limbs but also induces Hebbian plasticity. Research indicates that following long-term BSI training, residual neural fibers below the injury level exhibit enhanced functional connectivity even when the system is turned off, enabling the patient to perform a degree of autonomous walking with crutches. This finding challenges the traditional dogma that the CNS cannot regenerate, demonstrating that neural activity reinforced by electronic means can reverse pathological neural silence and promote synaptic reorganization at the biological level. To facilitate a clearer comparison of the translational landscape in neurotechnology-enabled SCI rehabilitation, we summarize representative advances from international and Chinese groups in Tables 4, 5 respectively to highlight the system architectures, clinical trial status, and reported functional outcomes (Trivano, 2025; Mertz, 2025; Jaszczuk et al., 2025; Oxley, 2024; Karami, 2024; Dohle et al., 2025; Xuanwu Hospital et al., 2023; Zhou and Yin, 2025; Available from, 2025; Li, 2025).

**TABLE 4:** Clinical applications in spinal cord injury: international progres**s**.

Institution	Product name	Trial ID (NCT)	Trial name	Technology type	Indication/Goal	Key status (2026)	References
Onward medical	ARC-BCI	NCT04632290	STIMO-BSI	Semi-invasive (WIMAGINE + ARC-IM)	Restoration of walking and upper-limb function after spinal cord injury	Recruiting/ongoing. Selected into the FDA TAP program; multiple patients implanted; effects are significant.	(Mertz, 2025)
Onward medical	ARC-IM	NCT07147296	Empower BP	Implantable stimulation (spinal cord end only)	Blood pressure instability caused by SCI (autonomic nervous function)	Ongoing. Verified the safety of the spinal cord stimulation hardware.	(Trivano, 2025)
Neuralink	N1 implant	NCT06429735	PRIME study	Fully invasive (1024 channels)	Tetraplegia (cursor control) - > motor recovery	Ongoing. The first patient set a record for cursor-control speed; expanded to Canada (CAN-PRIME).	(Jaszczuk et al., 2025)
Synchron	Stentrode	NCT05035823	COMMAND	Endovascular minimally invasive	Severe paralysis (digital device control)	Pivotal trial in preparation. Early feasibility studies completed; safety data are excellent.	(Oxley, 2024)
Blackrock Neurotech	MoveAgain	NCT01364480	BrainGate2	Fully invasive (Utah array)	Tetraplegia (robotic arm/FES control)	Long-term ongoing. FDA breakthrough device designation.	(Karami, 2024)
Paradromics	Connexus	(IDE approved)	Connect-one	Fully invasive (microneedle array)	Severe motor/speech impairment (communication restoration)	Recruiting (launched in 2025).	(Dohle et al., 2025)

ARC-BCI Adaptive/Active/ARC brain–computer interface (product name), ARC-IM ARC implantable stimulator/modulator (product name), BCI brain–computer interface, BP blood pressure, CAN-PRIME Canadian PRIME study extension (Canada arm of PRIME), FES functional electrical stimulation, FDA (U.S.) food and drug administration, IDE investigational device exemption, NCT National Clinical Trial (ClinicalTrials.gov identifier), SCI spinal cord injury, NIs neural interfaces.

**TABLE 5:** Clinical applications in spinal cord injury: progress in China.

Institution	System name	Registration/Trial ID	Technology type	Key features	Clinical progress (2021–2026)	References
Tsinghua University	NEO system	NCT05920174	Semi-invasive (wireless epidural)	Battery-free implant; coin-sized device(s); pneumatic glove assistance.	Successful. The first C4 tetraplegia patient achieved brain-controlled drinking and improved sensation; validated for home use.	(A prospective clinical trial, 2023)
Fudan University	Brain–spinal interface	NA	Invasive (cortical + spinal cord stimulation)	Millisecond-level low-latency AI algorithm; designed for complete spinal cord injury.	Breakthrough. By March 2025, the 4th case was completed; the first complete SCI patient regained walking.	(Zhou and Yin, 2025)
Wuhan University	Spine–machine interface	NA	Implantable (lumbosacral enlargement spinal cord chip)	Focused on spinal cord modulation; treats paralysis and refractory pain.	Scaled implementation. Completed 30+ tetraplegia and 100+ hemiplegia surgeries; leading clinical volume.	(Wuhan University Zhongnan hospital, 2025)
Tianjin University	ShenGong–HuaTuo	NA	Non-invasive	Brain-controlled acupuncture; wearable; rehabilitation training.	Deployed. Implemented in multiple hospitals; released the “ShenGong–HuaTuo” platform.	(Li, 2025)

AI, artificial intelligence, C4 cervical spinal cord level 4, NA, not available, NCT, National Clinical Trial (ClinicalTrials.gov identifier), SCI, spinal cord injury, NIs, neural interfaces.

##### Stroke

Although the primary lesion in stroke is cerebral, the resulting hemiplegia is fundamentally a disruption of descending control pathways. The application of NIs in stroke rehabilitation focuses on overcoming the therapeutic plateau often encountered in traditional physical therapy by enhancing the compensatory capacity of ipsilateral pathways or modulating the excitability of spinal reflexes.

Addressing persistent upper limb deficits post-stroke, the IpsiHand brain-computer interface system proposes a groundbreaking “ipsilateral control” strategy (Rustamov et al., 2025). Unlike traditional rehabilitation theories that focus on repairing the damaged contralateral corticospinal tract, the IpsiHand system leverages the intact contralesional hemisphere (Rustamov et al., 2023). By detecting motor intention signals (specifically, μ-rhythm desynchronization) via electroencephalography from the healthy hemisphere, the system drives an exoskeleton worn on the affected hand to complete grasping movements. This strategy activates the uncrossed anterior corticospinal tract and the reticulospinal tract. Clinical data show that after 12 weeks of home-based training, chronic stroke patients improved their Fugl-Meyer Assessment for Upper Extremity (FMA-UE) scores by an average of 8.1 points. This result far exceeds the minimal clinically important difference, confirming the neuroanatomical basis for using the healthy hemisphere to compensate for affected functions.

Another technology garnering significant attention is VNS paired therapy (Andalib et al., 2023). Its core mechanism relies on the temporal gating effect of neuromodulators. VNS triggers the release of NE from the locus coeruleus and acetylcholine from the basal forebrain, two key neurotransmitters regulating synaptic plasticity. When VNS is synchronized within hundreds of milliseconds of a specific rehabilitation movement (such as attempting to grasp), the brain tags the action as a “salient event” and specifically strengthens the associated neural circuits (Liu et al., 2023b; Wang et al., 2025b). Data from the pivotal Phase III VNS-REHAB trial and another meta-analysis demonstrate that this paired therapy not only significantly improved the upper limb function during the treatment period but also maintained the efficacy 1 year post-treatment. Accompanied by marked improvements in quality of life, this therapy has established itself as a gold standard in chronic stroke rehabilitation (Zhang et al., 2020; Dawson et al., 2021; Ramos-Castaneda et al., 2022; Liu et al., 2022a; Ananda et al., 2023; Zhou et al., 2025; Liu et al., 2022b; Yu and Wang, 2026).

Regarding lower limb rehabilitation, SCS has been innovatively applied to improve hemiplegic gait, leveraging insights from the SCI field. The FAST Walk system developed in Japan represents the forefront of this direction (Usman et al., 2026). Unlike continuous stimulation, this system employs an EMG-triggered mechanism that delivers transcutaneous ES to the T11-T12 spinal segments only when weak residual muscle activity is detected in the affected limb. This strategy ingeniously harnesses endogenous spinal reflex mechanisms (such as the crossed extensor reflex) to enhance propulsion in the affected limb and coordinate support in the healthy limb, significantly increasing gait speed and reducing energy expenditure. Additionally, Implanted Epidural Electrical Stimulation (EES) has proven to possess unique anti-spasticity effects (de Seta and Romeni, 2025; Powell et al., 2023). By using high-frequency stimulation to inhibit the hyperexcitability of spinal reflex arcs, EES effectively ameliorates spastic movement disorders common in stroke patients, such as the scissors gait.

#### Applications in peripheral nerve injuries

Although the peripheral nervous system possesses intrinsic regenerative capacity, natural repair processes are often slow and incomplete in cases of long-gap defects or complex injuries. Consequently, applications of bioelectronic medicine in this field are shifting from passive physical bridging toward the active induction of regeneration and high-fidelity functional reconstruction.

##### Peripheral nerve regeneration and repair

While traditional nerve guidance conduits (NGCs) merely provide physical support, next-generation bioelectronic scaffolds aim to actively modulate the molecular biological processes underlying regeneration through electrical signals.

Recent studies have elucidated the mechanisms by which ES drives nerve regeneration at the cellular and molecular levels. ES has been demonstrated to specifically activate the MAPK/ERK and PI3K/Akt signaling pathways, which are pivotal hubs regulating Schwann cell dedifferentiation, proliferation, and myelination (Thakkar et al., 2025). The application of ES with specific parameters during the acute phase of injury can significantly upregulate the expression of BDNF and Glial Cell Line-Derived Neurotrophic Factor (GDNF) at the injury site, providing essential trophic support for axonal extension. Furthermore, ES exhibits unique immunomodulatory capabilities, promoting the polarization of macrophages from the pro-inflammatory M1 phenotype to the reparative M2 phenotype. This creates an anti-inflammatory and pro-regenerative microenvironment, thereby reducing scar formation and accelerating tissue remodeling (Farahani and Shafiee, 2021; Li et al., 2024a; Jiang et al., 2023).

To facilitate long-term *in vivo* modulation, Smart NGCs integrate conductive polymers (such as polypyrrole and polyaniline) with 3D-printed biomimetic topological structures (Wu et al., 2025b; Zhang et al., 2025b; Lam et al., 2025; Li et al., 2025c). These conduits not only restore the endogenous bioelectric fields between damaged nerve stumps but also possess environment-responsive capabilities, enabling on-demand drug release based on local inflammatory factor concentrations. Regarding power supply, self-powered systems based on triboelectric nanogenerators (TENGs) and piezoelectric materials have achieved significant breakthroughs (Sun, 2025). By harvesting mechanical energy from respiration or muscle contractions to power implants, these systems realize a wireless, battery-free “electronic bandage” therapeutic approach, which drastically reduces infection risks and extends device longevity.

##### Motor output reconstruction and closed-loop FES

In cases where nerve transection leads to muscle denervation or amputation, re-establishing high-fidelity communication between the brain and effectors is critical for functional recovery.

Regenerative Peripheral Nerve Interface (RPNI) technology represents a seamless fusion of microsurgery and neural engineering (Burke et al., 2022). By implanting transected peripheral nerve fascicles into small autologous muscle grafts, RPNI utilizes muscle as a natural “biological amplifier.” The electromyographic (EMG) signals (mV level) generated when neural impulses trigger graft contraction are hundreds of times larger than the original neural signals (μV level), which effectively resolves the challenge of low signal-to-noise ratios (Wang et al., 2025e). Leveraging fascicular selectivity, surgeons can dissect large nerves into multiple independent control units to separately drive different fingers of multi-degree-of-freedom (DOF) dexterous prostheses, achieving highly intuitive motor control (Berberoglu et al., 2024). Furthermore, RPNI provides physiological targets for transected axons, effectively inhibiting the formation of painful neuromas and significantly alleviating phantom limb pain (Kubiak et al., 2022; González-Prieto et al., 2024; Hooper et al., 2020; Dehdashtian et al., 2023).

FES targeting paralyzed muscles is transitioning from open-loop to closed-loop paradigms. Novel closed-loop FES systems integrate joint-angle sensors and inertial measurement units to adjust stimulation parameters in real time according to gait phase or muscle fatigue. MPC is attractive because it can predict fatigue-related force decline and explicitly incorporate actuator and safety constraints. However, MPC requires repeated optimization at each control step, and computational latency increases with physiological model complexity, prediction horizon, and the number of stimulation channels. Reduced-order models, shorter prediction horizons, explicit or approximate MPC, Koopman-based linear representations, and hardware acceleration can reduce computational burden while preserving clinically meaningful control performance (Singh et al., 2025; Begon et al., 2025; Cavalcanti et al., 2025; Coelho-Magalhães et al., 2025; Vis, 2025). Computational modeling also provides an important layer of design and safety assessment before closed-loop control is implemented. Realistic peripheral nerve models indicate that electrode spacing, geometry, tissue conductivity, and the extent of nerve damage can substantially alter current distribution and neural recruitment (Du et al., 2021; Du et al., 2023b; Du et al., 2022). Integrated computational and experimental bioelectromagnetic frameworks can link field solutions to neuronal excitation and guide neuroprosthetic design (Stang et al., 2019). Machine-learning analysis can additionally detect complex, non-Gaussian morphological changes after peripheral nerve stimulation and support longitudinal safety assessment (Morales et al., 2024). More broadly, parameter-sweep optimization has been used in targeted nasal-spray delivery to identify multivariable operating windows (Du et al., 2023a); although that application is not neural stimulation, it illustrates a transferable strategy for jointly optimizing device geometry and delivery parameters under practical constraints. These models complement, rather than replace, *in vivo* validation and should not be treated as sole safety criteria.

Addressing specific issues such as foot drop, closed-loop systems precisely trigger stimulation during the swing phase and deactivate it during the stance phase, thus achieving more natural and energy-efficient gait assistance (Gather et al., 2025; He et al., 2025a; He et al., 2025b).

##### Sensory pathway reconstruction and modulation

Complementing motor output reconstruction, restoring and stabilizing afferent pathways is equally essential for functional integration and long-term recovery. The absence of sensory feedback is a primary barrier to prosthetic “embodiment,” while intractable neuropathic pain remains a persistent challenge for PNI patients (Zbinden et al., 2022; Donati and Valle, 2024; Finnerup et al., 2021). Bioelectronic systems provide a solution by reshaping sensory pathways (Dong et al., 2024).

The Dermal Sensory Regenerative Peripheral Nerve Interface (DS-RPNI) mirrors the RPNI used for motor control (Wang et al., 2025e; Chen et al., 2026b; Tian et al., 2025; Pettersen, 2025). In this approach, sensory nerve fascicles are implanted into de-epithelialized dermal grafts. Regenerating sensory nerve endings form mechanoreceptor structures within the dermal matrix. When the external devices apply pressure or ES to the graft, patients perceive tactile sensations or even texture. This bidirectional interaction not only endows the prosthesis with a “sense of touch” but also enhances the brain’s sense of ownership over the device through matched visuo-tactile feedback, thereby promoting psychological rehabilitation.

The efficacy of PNS for neuropathic pain is limited by stimulation instability. The latest closed-loop PNS systems introduce the Evoked Compound Action Potential (ECAP) as a feedback variable (Jekal et al., 2025). Immediately after delivering a stimulation pulse, the system records the ECAP amplitude to assess the activation level of nerve fibers. When changes in body posture alter the electrode-nerve distance, the system automatically adjusts the current intensity within milliseconds, ensuring that the stimulation remains within the optimal therapeutic window. Clinical trials have demonstrated that this closed-loop mechanism significantly increases the Responder Rate and eliminates the issues of over- or under-stimulation common encountered in open-loop systems (Eshraghi et al., 2026). Additionally, non-invasive Photobiomodulation Therapy (PBMT) utilizes near-infrared light to modulate mitochondrial function and inhibit neuronal hyperexcitability, offering a non-invasive adjunct therapy for pharmacoresistant pain (Liu et al., 2025b; Mauriello et al., 2025).

For functional musculoskeletal disorders, the key distinction among current strategies is whether the interface substitutes for missing neural commands, adapts residual circuits, or promotes biological reconnection. Brain–spine interfaces and closed-loop FES can provide rapid functional assistance but depend on reliable decoding and stimulation; paired VNS and EES rely more strongly on activity-dependent plasticity and may support durable gains, but require preserved neural substrates and intensive rehabilitation; regenerative peripheral interfaces can improve long-term biological integration, but involve surgery and slower tissue remodeling. Accordingly, no single architecture is universally optimal, and device selection should be guided by lesion level, residual neural function, therapeutic timescale, invasiveness, and the need for chronic implantation.

In summary, recent research progresses demonstrate that NIs and bioelectronic medicine have evolved from peripheral adjunctive technologies into core therapeutic pillars in the diagnosis and treatment of functional MSDs. From the “Digital Bridge” reconstruction and closed-loop autonomic management in the CNS to molecular regeneration induction and high-fidelity functional reanimation in the PNS, these technologies have profoundly reshaped our understanding of the repair capacity of the neuromusculoskeletal system.

Collectively, bioelectronic systems are evolving along a continuum: from open-loop actuation, to closed-loop control, and further toward autonomous closed-loop operation that can self-tune across contexts and time. For clarity, we refer to this autonomy as “self-decision” in the following sections. This shift raises an enabling question: what materials and architectures are required to sustain high-fidelity sensing, on-device computation, and adaptive intervention under mechanical deformation and long-term bio integration constraints?

## Enabling materials and system architectures for self-decision musculoskeletal NIs

Building on the above-mentioned three principles of closed-loop integration, regenerative modulation and seamless fusion, we next address the enabling question: what allows for a musculoskeletal neural interface to operate in a truly self-decision manner? Here, self-decision is not merely an algorithmic concept; it requires materials and architectures that sustain high-fidelity sensing, on-device computation, and adaptive intervention under mechanical deformation and long-term biofluid exposure. In this section, we focus on the material foundations and structural engineering strategies that mitigate mechanical/electrochemical mismatch and support durable closed-loop performance (Figure 7).

**FIGURE 7:**
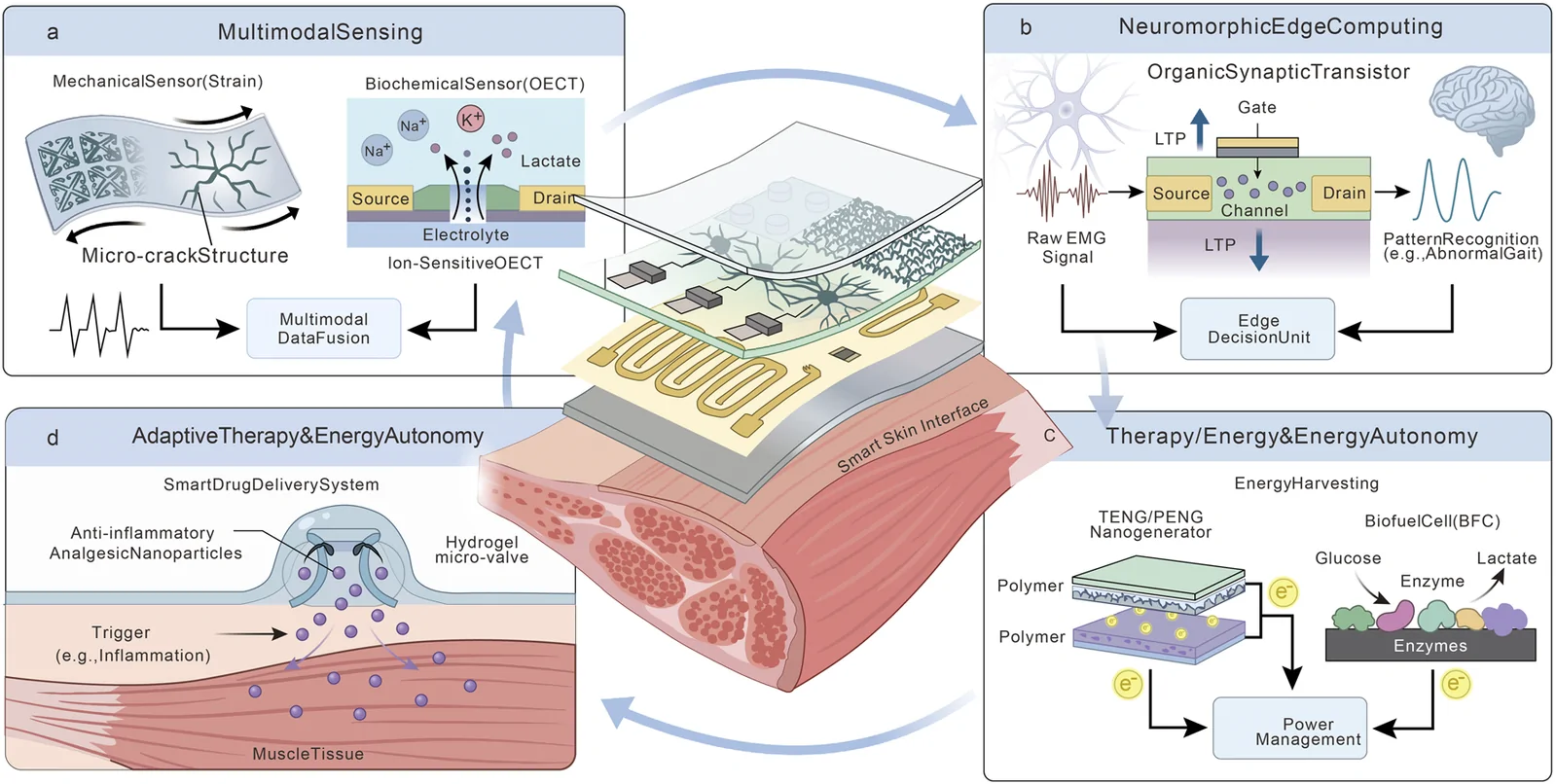
Materials and system design for self-decision musculoskeletal neural interfaces. **(a)** Multimodal sensing integrates strain-based mechanical sensors (e.g., micro-crack structures) with biochemical monitoring via ion-sensitive OECTs, followed by multimodal data fusion. **(b)** Neuromorphic edge computing uses organic synaptic transistors to realize in-memory processing and synaptic plasticity (LTP/LTD), enabling on-device EMG pattern recognition (e.g., abnormal gait) and edge decision-making. **(c)** Energy autonomy is supported by mechanical energy harvesters (TENG/PENG) and biofuel cells (BFCs) coupled with power management. **(d)** Adaptive therapy employs smart drug-delivery modules (e.g., hydrogel micro-valves) for trigger-responsive release of anti-inflammatory/analgesic agents, completing a closed-loop sensing–decision–intervention architecture. Abbreviations: BFC biofuel cell, EMG electromyography, LTD long-term depression, LTP long-term potentiation, OECT organic electrochemical transistor, PENG piezoelectric nanogenerator, TENG triboelectric nanogenerator.

### Material paradigms for self-decision

Traditional diagnostic and therapeutic models are constrained by the latency between measurement and intervention, rendering them ill-suited to address the dynamic fluctuations inherent in musculoskeletal pathologies, such as muscle spasticity or loss of motor function. Consequently, next-generation NIs aim to construct a unified, closed-loop architecture that seamlessly integrates sensing, decision-making, and treatment. To achieve this objective, material selection is no longer dictated solely by conductivity or mechanical strength; rather, it has evolved into a chemical and structural language capable of facilitating signal transduction across the soft-hard interface.

#### Selection of material categories

Conductive polymers (such as PEDOT:PSS, polyaniline, and polypyrrole) have emerged as pivotal bridges connecting organic soft tissues with electronic functions, owing to their combined intrinsic electronic conductivity, flexibility, and processability (Hasan et al., 2025). Pure PEDOT:PSS hydrogels, in particular, stand out by exhibiting a conductivity as high as ∼286 S/cm (Oh et al., 2024). Furthermore, by forming interpenetrating networks, they significantly reduce interfacial impedance, thus effectively overcoming the signal transmission barriers inherent to traditional metal electrode-neural tissue interfaces (Kim et al., 2025).

In addition, carbon nanomaterials (e.g., carbon nanotubes and graphene) have been extensively employed in constructing high signal-to-noise ratio (SNR) neural recording electrodes (McCallum et al., 2017), leveraging their exceptional mechanical strength and high carrier mobility (Hu et al., 2024; Peng et al., 2023; Chen et al., 2024). Carbon nanotubes (CNTs) can specifically augment the effective electrochemical surface area and form highly conductive percolation networks (Vafaiee et al., 2020). This mechanism lowers charge transfer resistance and enhances capacitance, which is critical for maintaining signal stability in long-term implantation environments.

To guarantee long-term biocompatibility and attenuate immune rejection, the application of bio-based and biodegradable materials (such as gelatin, chitosan, and polylactic acid) is gaining increasing prevalence (Ren et al., 2025; Heras-Mozos et al., 2022; Guicherd et al., 2024). These materials offer abundant chemical functional groups (e.g., amine and carboxyl groups) for bioconjugation and can form hydrogel scaffolds that mimic the extracellular matrix (ECM), thereby supporting soft tissue integration. Hybrid composites, constructed by embedding conductive components within biodegradable matrices, represent an ideal paradigm for achieving the long-term stable operation of NIs. They successfully retain tissue-like softness while establishing efficient networks for charge or ion transport.

#### Construction of material structures

The performance envelope of neural interfaces targeting the musculoskeletal system is not determined by a single scale but is the result of the hierarchical coupling of atomic, molecular, mesoscopic, and macroscopic structures (Liao et al., 2025).

At the mesoscale, the geometry (e.g., nanowires and nanotubes), alignment, and dispersion of fillers determine the percolation threshold and anisotropic conductivity (Li et al., 2024b). Given therapeutic actuation requiring high charge injection capacity (such as muscle ES), employing porous nanostructured coatings or fractal architectures can maximize the accessible electrochemical surface area. This significantly enhances capacitance without increasing the geometric dimensions (Viana et al., 2024). For example, hydrogel scaffolds with aligned nanopores exhibit superior performance in controlling diffusion rates and spatial selectivity compared to isotropically swelling bulk hydrogels (Lu et al., 2024).

At the macroscale, patterned architectures and gradient stiffness designs are crucial for regulating mechanical matching and strain distribution (Zhang et al., 2024). Addressing the large-strain environments common in the musculoskeletal system (e.g., joint movement), embedding percolative nanofiller networks into elastomeric matrices represents an effective strategy (Xu et al., 2024). By leveraging the elastic reconfiguration of the mesoscopic network to maintain conductive pathways, this approach achieves large-range strain sensing while preserving high sensitivity (Gauge Factor). Furthermore, to mitigate material degradation during long-term implantation, introducing redundant conductive pathways or self-healing networks (such as dynamic cross-linking-based polyborosiloxane-carbon nanotube composites) can endow the interface with the ability to rapidly recover conductivity following physical damage (Tang et al., 2025).

#### Interfacial integration with tissue

The central challenge of neural interfaces lies in optimizing the “bio-abiotic” interface to ensure efficient ion-electron signal transduction while minimizing mechanical mismatch (Li et al., 2024c). Traditional metal electrodes are often limited by high impedance and low charge injection capacity (Boehler et al., 2020; Duan et al., 2024). In contrast, conductive polymer coatings, such as PEDOT:PSS, can reduce the interfacial impedance of microelectrodes to approximately 1% of that of bare metal electrodes, while significantly boosting charge injection capacity (Teixeira et al., 2022; Dijk et al., 2023). This low-impedance characteristic directly translates into higher signal fidelity and reduced interfacial capacitive delay, which is critical for capturing fast-changing neuromuscular electrophysiological signals.

Given the soft and dynamic nature of musculoskeletal tissue, rigid electronic devices are prone to triggering inflammatory encapsulation or interfacial delamination. Employing ultrathin elastomeric films or hydrogels as substrates can drastically reduce the Young’s modulus mismatch (Tringides et al., 2021; Mariello et al., 2022). For instance, encapsulating conductive carbon nanomaterial arrays within viscoelastic hydrogels can form a plastically deformable interface that establishes conformal contact with soft tissue geometry, thereby buffering mechanical stress. Additionally, surface modification strategies—such as using zwitterionic polymer brushes to achieve anti-biofouling properties (Yang et al., 2020), or utilizing functional groups like catechol and silane to enhance interfacial adhesion—are essential for maintaining long-term implantation stability (Xie et al., 2020).

To protect electronic components within the complex *in vivo* ionic environment, encapsulation systems must combine hermeticity with mechanical compliance (Nanbakhsh et al., 2025). Liquid metal-based elastic sealing strategies, along with multilayer barrier stacks composed of inorganic Atomic Layer Deposition (ALD) layers and organic polymers (e.g., Parylene), have demonstrated the potential to block ion permeation under dynamic deformation (Shen et al., 2023). This “universal encapsulation” strategy employs gradient modulus transitions to effectively prevent delamination between rigid chips and soft substrates, thus ensuring the mechanical integrity of the system during large-amplitude musculoskeletal movements (Shao et al., 2024).

A major translational limitation is that favorable acute electrochemical properties do not necessarily persist during chronic implantation. Protein adsorption, inflammatory or fibrotic encapsulation, and increasing electrode–tissue separation can raise interfacial impedance and attenuate recorded neural signals, progressively reducing SNR. Repetitive micromotion may induce microcracks or delamination of conductive coatings, while prolonged exposure to ionic biofluids can accelerate swelling, corrosion, and encapsulation failure. For stimulation electrodes, repeated charge transfer may reduce the effective electrochemical surface area or destabilize high-capacitance coatings, thereby decreasing usable charge injection capacity and narrowing the safe stimulation window. Long-term evaluation should therefore integrate impedance drift, SNR, charge injection capacity, mechanical integrity, and histological tissue response rather than relying only on initial conductivity or bench-top electrochemical measurements.

### Multimodal sensing and in-situ biochemical monitoring

To precisely capture the complex pathological states of the musculoskeletal system, ideal neural interfaces must transcend single-modality electrophysiological recording and evolve into “smart skins” capable of multimodal sensing (Ates et al., 2022; Yuan and Shen, 2023). Morphologically, such interfaces are no longer simple arrays of metallic contacts but flexible composites integrating physical and biochemical sensors, capable of synchronously decoding mechanical motion and metabolic microenvironments (Wang et al., 2022; Spinelli et al., 2025).

#### Mechanical sensors

Targeting the unique mechanical behaviors of the musculoskeletal system, mechanical sensors constitute the physical sensing layer of the interface. Flexible strain sensors based on piezoresistive, capacitive, or triboelectric mechanisms are seamlessly integrated into the device substrate. By monitoring the dynamic response of resistance or capacitance to geometric deformation, these sensors achieve high-precision tracking of joint angles, muscle contraction intensity, and gait patterns (Meng et al., 2025). To achieve high sensitivity (high Gauge Factor) within a wide linear range, material designs often employ micro-crack structures, fractal geometric patterns, or porous conductive networks. This ensures that the device maintains signal linearity and low hysteresis even during large-scale skeletal movements (Zahri et al., 2024).

#### Biochemical sensors

More critically, next-generation interfaces must possess the capability to gain insight into tissue metabolic states. The introduction of biochemical sensors enables the real-time monitoring of ion concentrations (e.g., Na+, K+ and Ca2+), metabolites (e.g., lactate and glucose), and neurotransmitter fluctuations, which is vital for the early warning of muscle fatigue or inflammatory responses (Gao et al., 2023). For instance, ion-sensitive receptors developed by using organic electrochemical transistors (OECTs) leverage their high transconductance to convert weak ion fluxes into significant current signals. This enables high-sensitivity *in-situ* detection of pH values or specific biomarkers within the muscle microenvironment (Lu et al., 2025). Fusion of multimodal data, known as synchronous acquisition of “electrical-mechanical-chemical” signals, provides full-dimensional data support for constructing high-fidelity disease models.

Multimodal sensing becomes clinically meaningful only when heterogeneous measurements are converted into physiologically interpretable state variables. EMG can characterize muscle activation; IMUs and joint-angle sensors can identify limb kinematics and gait phase; and strain or force sensors can estimate tissue loading. Fusion of these signals can generate latent biomechanical states such as swing or stance phase, contraction quality, joint loading, and fatigue, which are more directly actionable for closed-loop control than any single raw signal. Biochemical measurements of lactate, pH, ions, or inflammatory mediators provide a slower-timescale description of metabolic or inflammatory state and can complement fast electrophysiological and mechanical feedback. Effective fusion requires temporal synchronization, calibration across modalities, drift compensation, and confidence-aware algorithms that account for differences in sampling rate and signal reliability. The resulting state estimates can then be mapped to stimulation timing, amplitude, pulse width, or therapeutic mode, completing the transition from multimodal acquisition to biomechanically informed adaptive control.

## Neuromorphic computing and edge decision architecture

In traditional architectures, large numbers of analog signals collected by sensors must be transmitted to external hardware for digitization, which not only incurs huge power consumption but also introduces non-negligible latency, severely constraining real-time intervention for sudden events such as muscle spasms (Xia Z. et al., 2025). Consequently, advanced neural interfaces targeting the musculoskeletal system are undergoing a paradigm shift in architecture by moving from the traditional “von Neumann” segregated architecture toward brain-inspired neuromorphic computing (Boahen et al., 2025).

Physically, these novel interfaces manifest as “computing-in-memory” units that integrate sensing, computing, and storage. By utilizing memristors or synaptic transistors to mimic the plasticity of biological synapses, these devices can process sensory signals directly in the analog domain. This allows for *in-situ* signal denoising, feature extraction, and even pattern recognition (Herbozo Contreras et al., 2024; Yuan et al., 2023; Janzakova et al., 2023). For instance, conjugated polymer-based organic synaptic transistors can regulate conductance weights via the doping/dedoping states of ions within the channel, simulating Long-Term Potentiation (LTP) and Long-Term Depression (LTD) of synaptic weights. This material-level memory and learning capability empowers the interface to directly recognize specific electromyography (EMG) patterns or abnormal gaits without the need to upload raw data to the cloud (Ji et al., 2025). This “edge computing” capability enables the neural interface to function as an independent agent, making self-decisions regarding pathological signals at millisecond speeds. This drastically reduces system power consumption and enhances the real-time performance of closed-loop control (Lee et al., 2021; Xu J. et al., 2025).

Algorithm selection is a central constraint for closed-loop neural interfaces operating under limited on-device power, memory, and computational resources. Lightweight state-space models, shallow neural networks, compact convolutional architectures, and spiking or neuromorphic models are generally more compatible with edge implementation than large deep networks that require substantial memory movement and multiply–accumulate operations. Architecture selection should therefore consider inference latency, memory footprint, energy per decision, and the required control-loop update rate in addition to predictive accuracy. In FES, delayed decisions can reduce phase-specific stimulation accuracy or destabilize movement; closed-loop accuracy must consequently be optimized jointly with latency and power consumption.

### Adaptive therapeutic execution and self-powered operation

The ultimate goal of “self-decision” systems is to execute precise therapeutic interventions based on the outcomes of sensing and computing. Therefore, an ideal neural interface must structurally incorporate controllable actuation modules. Beyond traditional FES electrodes, smart drug delivery systems capable of responding to specific physical or chemical triggers are emerging as integral components of the interface (Mage et al., 2017). By leveraging the redox-induced volumetric expansion/contraction of conductive polymer films, or the stimuli-responsive swelling/deswelling mechanisms of hydrogels, interfaces can construct micropumps or smart valves (Beck et al., 2023; Arango et al., 2020; Gupta et al., 2021). When the computing unit determines that an inflammation flare-up has occurred or a pain threshold has been breached, these actuators can release anti-inflammatory drugs or analgesics on-demand, achieving a fully closed loop of “detection-diagnosis-treatment (Liu et al., 2024; He et al., 2024).”

Finally, to liberate the system from bulky batteries and adapt to the long-term implantation requirements of the musculoskeletal system, energy autonomy represents the final piece of the system design puzzle (Khan et al., 2024; Jiang et al., 2020). Ideal interfaces should possess the capability to harvest energy from their surrounding environment. Triboelectric Nanogenerators (TENGs) and Piezoelectric Nanogenerators (PENGs) harvest the abundant mechanical energy generated by musculoskeletal movement. By converting this energy into electricity through contact-separation or lattice polarization modes, they not only power low-power neuromorphic chips but can also serve as Direct Electrical Stimulation (DES) sources themselves to promote tissue regeneration or modulate neural activity (Yao et al., 2021; Liu et al., 2022c). Furthermore, for deep implantation scenarios, enzyme- or microbe-based BFCs utilize glucose or lactate in body fluids as fuel, which provides another sustainable biochemical energy solution.

In summary, neural interfaces targeting MSDs are evolving from single electrode probes into highly integrated, soft-matter smart microsystems. They mimic the extracellular matrix (ECM) in material composition, adapt to dynamic mechanical environments in structure, and replicate the sensing-computing-responding loop of the biological nervous system in function. This system design, which deeply integrates materials science, electronic engineering, and biomedicine, constitutes the material foundation for realizing personalized and precise diagnosis and treatment of MSDs in the future.

## Summary and outlook

The advent of neural interface technologies marks a profound paradigm shift in the treatment of MSDs. We are transitioning from a “mechanical era,” dominated by anatomical structural replacement, toward a “bioelectronic era” centered on neuromodulation and tissue regeneration. As highlighted throughout this review, the nervous system acts not merely as a passive conduit for motor commands but as a “master regulator” maintaining musculoskeletal metabolic homeostasis, the immune microenvironment, and regenerative capacity. High-precision neural interfaces enable us not only to restore motor function in paralyzed limbs and may modulate pathological processes of osteoporosis, sarcopenia, and chronic inflammation by modulating the “neuro-bone axis” or the local neural microenvironment. This dual therapeutic efficacy by encompassing both “functional substitution” and “disease modification” underscores the immense potential of bioelectronic medicine in the field of MSDs. However, translating laboratory breakthroughs into widespread clinical therapies requires bridging the persistent gap between biological mechanisms and engineering technologies. Future research should prioritize breakthroughs in the following key directions:

Current neuromodulation strategies predominantly rely on simple ES waveforms, such as constant-frequency pulses. In contrast, endogenous neural regeneration signals often contain complex spatiotemporal coding. A core challenge for the future lies in utilizing high-throughput electrophysiological recording and multi-omics technologies to decipher the specific “neural codes” that induce bone regeneration, vascularization, and muscle fiber maturation. For instance, it is critical to elucidate how stimulation at distinct frequencies specifically regulates the differentiation fate of stem cells, or how specific stimulation patterns can inhibit nociception while preserving proprioception. AI-based decoding algorithms will play a pivotal role in this process, aiding in the establishment of a precise mapping atlas between “electrical signals and biological effects.”

Existing neural interfaces are mostly open-loop systems or simple closed-loop systems based solely on electrophysiological signals (e.g., ECAP). However, the pathological states of MSDs involve complex biochemical alterations. Next-generation interfaces should integrate multimodal sensing capabilities, enabling not only the perception of electrical signals but also the real-time monitoring of ion concentrations (Ca^2+^, K^+^), inflammatory cytokines (IL-6, TNF-α), and metabolites (lactate, pH) within the local microenvironment. Based on this multidimensional data, we can construct a true “bio-electronic closed-loop system” that dynamically adjusts stimulation strategies according to the real-time metabolic demands of the tissue, thereby realizing a leap from “on-demand stimulation” to “intelligent regeneration.”

The musculoskeletal system is a dynamic tissue environment subjected to continuous high-intensity mechanical loading. Traditional rigid electronic devices are prone to inducing immune rejection or mechanical failure following long-term implantation. Future materials science should focus on developing flexible electronic materials with Young’s moduli that match those of musculoskeletal tissues, as well as self-healing hydrogel interfaces and bioactive coatings. Furthermore, for temporary therapeutic needs such as fracture healing, “Transient Electronics” based on materials like magnesium (Mg) and molybdenum (Mo)—which degrade completely *in vivo* after fulfilling their therapeutic mission—represent a highly clinically valuable development direction.

With continued advances in neuroscience, materials science, and intelligent control, neural interfaces may become increasingly important components of personalized musculoskeletal care. However, broad clinical adoption will depend on demonstrating durable efficacy, long-term safety, manufacturing reproducibility, acceptable cost, and clear advantages over established pharmacological, surgical, and rehabilitation strategies.

## Glossary

**α7nAChR**: Alpha-7 nicotinic acetylcholine receptor
**ACh**: Acetylcholine
**AChR**: Acetylcholine receptor
**AChE**: Acetylcholinesterase
**ACR**: American college of rheumatology
**AI**: Artificial intelligence
**ALD**: Atomic layer deposition
**ALS**: Amyotrophic lateral sclerosis
**AP**: Action potential
**AR**: Adrenergic receptor
**ARC**: Arcuate nucleus
**BBB**: Blood–brain barrier
**BDNF**: Brain-derived neurotrophic factor
**BFC**: Biofuel cell
**BSI**: Brain–spine interface
**CDKN**: Cyclin-dependent kinase inhibitor 1a
**CGRP**: Calcitonin gene-related peptide
**CHSY**: Chondroitin sulfate synthase
**CLBP**: Chronic low back pain
**CNS**: Central nervous system
**CNT**: Carbon nanotube
**COX**: Cyclooxygenase
**CREB**: Camp response element-binding protein
**CRH**: Corticotropin-releasing hormone
**DES**: Direct electrical stimulation
**DMOAD**: Disease-modifying osteoarthritis drug
**DOF**: Degree(s) of freedom
**DRG**: Dorsal root ganglion
**DS**: Dermal sensory
**ECAP**: Evoked compound action potential
**ECM**: Extracellular matrix
**EES**: Epidural electrical stimulation
**EFNEM**: Electro-responsive functional neurovascularized engineered muscle
**EMG**: Electromyography
**EP**: E prostanoid receptor
**ERK**: Extracellular signal-regulated kinase
**ES**: Electrical stimulation
**EV**: Extracellular vesicle
**FAP**: Fibro-adipogenic progenitor
**FAST**: Fast-twitch muscle fiber type
**FDA**: U.s Food and drug administration
**FE**: Feature extraction
**FES**: Functional electrical stimulation
**FGF**: Fibroblast growth factor
**FGFR**: Fibroblast growth factor receptor
**FMA**: Fugl–meyer assessment
**GABA**: Gamma-aminobutyric acid
**GDNF**: Glial cell line-derived neurotrophic factor
**GPR**: G protein-coupled receptor
**IL**: Interleukin
**IM**: Implantable module
**IMU**: Inertial measurement unit
**IVD**: Intervertebral disc
**IVDD**: Intervertebral disc degeneration
**LC**: Locus coeruleus
**LMN**: Lower motor neuron
**LTD**: Long-term depression
**LTP**: Long-term potentiation
**MAPK**: Mitogen-activated protein kinase
**MPC**: Model predictive control
**MSC**: Mesenchymal stem cell
**MSD**: Musculoskeletal disorder
**NI**: Neural interface
**NE**: Norepinephrine
**NFAT**: Nuclear factor of activated t cells
**NGC**: Nerve guidance conduit
**NGF**: Nerve growth factor
**NMJ**: Neuromuscular junction
**OA**: Osteoarthritis
**OECT**: Organic electrochemical transistor
**OH**: Orthostatic hypotension
**PBMT**: Photobiomodulation therapy
**PEDOT**: Poly(3,4-ethylenedioxythiophene)
**PENG**: Piezoelectric nanogenerator
**PGE**: Prostaglandin e
**PNI**: Peripheral nerve injury
**PNS**: Peripheral nerve stimulation
**POMC**: Pro-opiomelanocortin
**PRG**: Proteoglycan
**PSCI**: Phase-locked spinal cord interface
**PSS**: Poly styrene sulfonate
**PVDF**: Poly vinylidene fluoride
**PVH**: Paraventricular hypothalamic nucleus
**RA**: Rheumatoid arthritis
**RAMP**: Receptor activity-modifying protein
**RANKL**: Receptor activator of nuclear factor kappa-b ligand
**RCAN**: Regulator of calcineurin 1
**REHAB**: Rehabilitation
**RNA**: Ribonucleic acid
**ROCK**: Rho-associated coiled-coil containing protein kinase
**ROS**: Reactive oxygen species
**RPNI**: Regenerative peripheral nerve interface
**SASP**: Senescence-associated secretory phenotype
**SCI**: Spinal cord injury
**SCS**: Spinal cord stimulation
**SNARE**: Soluble n-ethylmaleimide-sensitive factor attachment protein receptor
**SNR**: Signal-to-noise ratio
**SOD**: Superoxide dismutase
**TENG**: Triboelectric nanogenerator
**TGF**: Transforming growth factor
TKA: Total knee arthroplasty
**TNF**: Tumor necrosis factor
**TOR**: Target of rapamycin
**UE**: Upper extremity
**UMN**: Upper motor neuron
**VMH**: Ventromedial hypothalamus
**VML**: Volumetric muscle loss
**VNS**: Vagus nerve stimulation
